# DP103 as a critical modulator of Wnt signaling and cancer stemness: implications for precision treatment in triple negative breast cancer

**DOI:** 10.1038/s41419-026-08943-3

**Published:** 2026-06-05

**Authors:** Wanpei Cai, Sebastian Öther-Gee Pohl, Xianning Lai, Hiu Yan Lam, Catharine Shi-Yun Low, Vivien Koh, Haoran Ma, Mark Agostino, Arindam Banerjee, Nawat Bunnag, Jahnavi Suresh, Amudha Deivasigamani, Yi Yuan, Tuan Zea Tan, Muthukumar Rajasekaran, Jianxiang Chen, Hannah Lee Foon Swa, San Hue Hua, Shiyuan Guo, Janet Cheng-Fei Teng, Jas Min Chin, Jocelyn Yeen Hui Choy, Aye Aye Thike, Young Bok Lee, Madhu Mathi Kanchi, Karthik Ramesh, Yu Keung Mok, Ruby Yun-Ju Huang, Jayantha Gunaratne, Melissa Jane Fullwood, Puay Hoon Tan, George W. Yip, Gautam Sethi, Jeremy P. Blaydes, Celestial T. Yap, Peter Edward Lobie, Venetia Jing-Tong Kok, Arunasalam Dharmarajan, Kam Man Hui, Deog Joong Kim, Jit Kong Cheong, Nicholas Tolwinski, Wei Peng Yong, Patrick Tan, Kevin B. Myant, Alan Prem Kumar

**Affiliations:** 1 Department of Pharmacology, Yong Loo Lin School of Medicine, National University of Singapore, Singapore, Singapore; 2 NUS Centre for Cancer Research (N2CR), Yong Loo Lin School of Medicine, National University of Singapore, Singapore, Singapore; 3 Cancer Research UK Edinburgh Centre, Institute of Genetics and Cancer, The University of Edinburgh, Edinburgh, UK; 4 Department of Biochemistry, Yong Loo Lin School of Medicine, National University of Singapore, Singapore, Singapore; 5 National University Cancer Institute Singapore, National University Health System, Singapore, Singapore; 6 Cancer Science Institute of Singapore, National University of Singapore, Singapore, Singapore; 7 Program in Cancer and Stem Cell Biology, Duke-NUS Medical School, Singapore, Singapore; 8 Curtin Institute for Computation, Curtin University, Perth, Australia; 9 Cancer Sciences Unit, Faculty of Medicine, University of Southampton, Southampton, UK; 10 Biocon Limited, Bangalore, India; 11 Division of Science, Yale-NUS College, Singapore, Singapore; 12 Division of Cellular and Molecular Research, National Cancer Centre Singapore, Singapore, Singapore; 13 Programme in Cardiovascular & Metabolic Disorders, Duke-NUS Medical School, Singapore, Singapore; 14 Diagnostics Development Hub, A*STAR, Singapore, Singapore; 15 Department of Anatomy, Yong Loo Lin School of Medicine, National University of Singapore, Singapore, Singapore; 16 Division of Pathology, Singapore General Hospital, Singapore, Singapore; 17 Rexahn Pharmaceuticals Inc., Rockville, MD USA; 18 Department of Genome Sciences, The John Curtin School of Medical Research, The Australian National University, Canberra, ACT Australia; 19 Department of Biological Sciences, National University of Singapore, Singapore, Singapore; 20 School of Medicine, College of Medicine, National Taiwan University, Taipei, Taiwan; 21 Institute of Molecular and Cell Biology, A*STAR, Singapore, Singapore; 22 School of Biological Sciences, Nanyang Technological University, Singapore, Singapore, Singapore; 23 Luma Medical Centre, Singapore, Singapore; 24 Department of Physiology, Yong Loo Lin School of Medicine, National University of Singapore, Singapore, Singapore; 25 Tsinghua Berkeley Shenzhen Institute, Tsinghua University Graduate School at Shenzhen, Shenzhen, China; 26 Trichy SRM Medical College Hospital and Research Centre, Tiruchirappalli, India; 27 School of Human Sciences, Faculty of Life and Physical Sciences, The University of Western Australia, Perth, Australia; 28 Curtin Medical School, Curtin University, Perth, WA Australia; 29 Micro Tech Health Sdn Bhd, Kuala Lumpur, Malaysia; 30 Genome Institute of Singapore, Agency for Science, Technology and Research, Singapore, Singapore; 31 Singapore Gastric Cancer Consortium, Singapore, Singapore; 32 Singhealth/Duke-NUS Institute of Precision Medicine, National Heart Centre Singapore, Singapore, Singapore

**Keywords:** Biomarkers, Cancer

## Abstract

Triple-negative breast cancer (TNBC) is a clinically aggressive subtype lacking targeted therapies, often characterized by hyperactivation of the Wnt/β-catenin signaling pathway and an enriched population of cancer stem cells (CSCs). Here, we identify the DEAD-box RNA helicase DP103 as a novel modulator of Wnt/β-catenin signaling in TNBC, acting independently of its canonical helicase function. DP103 expression correlates with increased phosphorylation of LRP6 and nuclear β-catenin accumulation, enhancing Wnt transcriptional activity. Mechanistically, DP103 physically interacts with GSK3β to facilitate post-translational modifications essential for Wnt activation. Notably, DP103 itself is a Wnt target, forming a feedforward loop that sustains oncogenic signaling. Functional studies reveal that DP103 promotes CSC-like traits in TNBC cells, including self-renewal and expression of stemness markers (Nanog, Oct4, Sox2), linking its role in Wnt activation to breast cancer stemness and metastasis. In vivo studies using Drosophila models confirmed the evolutionarily conserved role of Gemin3/DP103 in epithelial transformation, though context-dependent differences were observed. Importantly, we demonstrate that RX-5902, a Wnt pathway inhibitor currently in clinical trials, suppresses DP103 expression and Wnt/β-catenin signaling, reducing TNBC cell viability and mammosphere formation without affecting normal epithelial cells. RX-5902 efficacy was abrogated by DP103 depletion, underscoring DP103’s critical role in mediating drug response. In xenograft models, RX-5902 treatment significantly reduced tumor burden and prolonged survival. Collectively, our findings establish DP103 as a key regulator of Wnt-driven oncogenesis in TNBC and highlight its dual role in promoting CSC traits and therapeutic resistance. These insights position DP103 as a potential biomarker and therapeutic target to disrupt sustained Wnt signaling in TNBC, offering new avenues for precision intervention in this challenging breast cancer subtype.

## Introduction

Triple-negative breast cancer (TNBC) is a highly heterogeneous disease characterized by multiple molecular subtypes, and typically presents with the loss of estrogen (ER), progesterone (PR), and human epidermal growth factor receptor 2 (HER2) expression [1]. Despite recent advances in breast cancer therapy, the prevalence and mortality of metastatic TNBC remain high [2]. Research suggests this may be due to a subset of quiescent, and drug-resistant cells, termed breast cancer stem cells (BCSCs) [3]. The prevalence of BCSCs across breast cancer subtypes is not equal. TNBC includes the basal-like and the claudin-low subtype, which are highly mesenchymal and display greater stem cell and epithelial–mesenchymal transition (EMT) characteristics that resemble human mammary stem cells [4, 5].

The Wnt/β-catenin signaling pathway is a multifaceted and evolutionarily conserved pathway that plays essential roles in early embryonic development and cell fate [6]; it has also been reported as one of the main pathways involved in the progression of TNBC [7]. Wnt signaling dysregulation is present in ~40% of all breast cancers [7–11]. Canonical Wnt signaling is primarily mediated through β-catenin-dependent mechanisms that are controlled through phosphorylation events by various kinases, including GSK3β and CK1α, regulating its degradation and nuclear localization. The basal-like 2 (BL2) and mesenchymal-like (ML) TNBC molecular subtypes exhibit gene signatures associated with increased activation of the Wnt signaling pathway [1]. Furthermore, BCSCs derived from TNBC cells display increased Wnt signaling [12], which is critical for their maintenance [9].

Overexpression of Wnt signaling components has also been reported in TNBC, although mutations in APC or β-catenin are rare [13–15]. Previously, targeting the canonical Wnt signaling pathway has had little success clinically. Clinical trials for Wnt/β-catenin targeted therapies such as Vantictumab (a Frizzled receptor mAb), ICG-001, and PRI-724 (inhibitors of β-catenin-transcriptional activating complexes) and LGK-974 (a Wnt ligand-specific acetyltransferase inhibitor) are ongoing, although further investigations to determine efficacy, specificity, patient safety, stratification, and delivery are still required [16, 17].

Overexpression of non-conventional Wnt regulators, such as the DEAD-box RNA helicase family member DDX5, further complicates Wnt/β-catenin signaling in TNBCs [18]. The lack of successful treatment options for TNBC and Wnt-driven TNBC exemplifies the need for the discovery of new drivers and potential therapeutic targets.

The DEAD/DExD box RNA helicases represent the largest group of the SF2 superfamily [19]. They are highly conserved enzymes containing domains within their helicase core with functional roles in ATP binding/hydrolysis and DNA/RNA helicase activity [20]. DEAD-box helicases have been widely studied for their biological roles in RNA metabolism [21–23], while their RNA metabolism-independent roles in modulating signaling pathways, including the Wnt signaling pathway, have only been recently described [24, 25]. DEAD-box family member, DP103 (Gemin3 or DDX20) functions in both RNA-dependent and independent roles [26]. It is associated with many cancers [27], where it can act as a tumor suppressor, regulating microRNA processing in hepatocellular carcinomas [28–31] or as an oncogene in inhibiting p53-mediated apoptosis in EBV-positive lymphoma cells [29]. These studies suggest the multifunctional role of DP103 in cancer, demonstrating both RNA-dependent and RNA-independent mechanisms.

DP103 was recently reported to be overexpressed in TNBC and was proposed as a prognostic marker for metastatic TNBC [32]. Here, we investigate DP103-mediated regulation of the Wnt/β-catenin pathway. Through its interaction with GSK3β, DP103 promotes Wnt/β-catenin-dependent cancer progression, metastasis, and regulates BCSCs in TNBC. The exclusive overexpression of DP103 and its interaction with GSK3β in the metastatic TNBC subtype make DP103 an attractive molecular target for therapeutics.

## Results

### Increased expression of DP103 and Wnt proteins in TNBC correlates with increased Wnt/β-catenin signaling activity

**Fig. 1:**
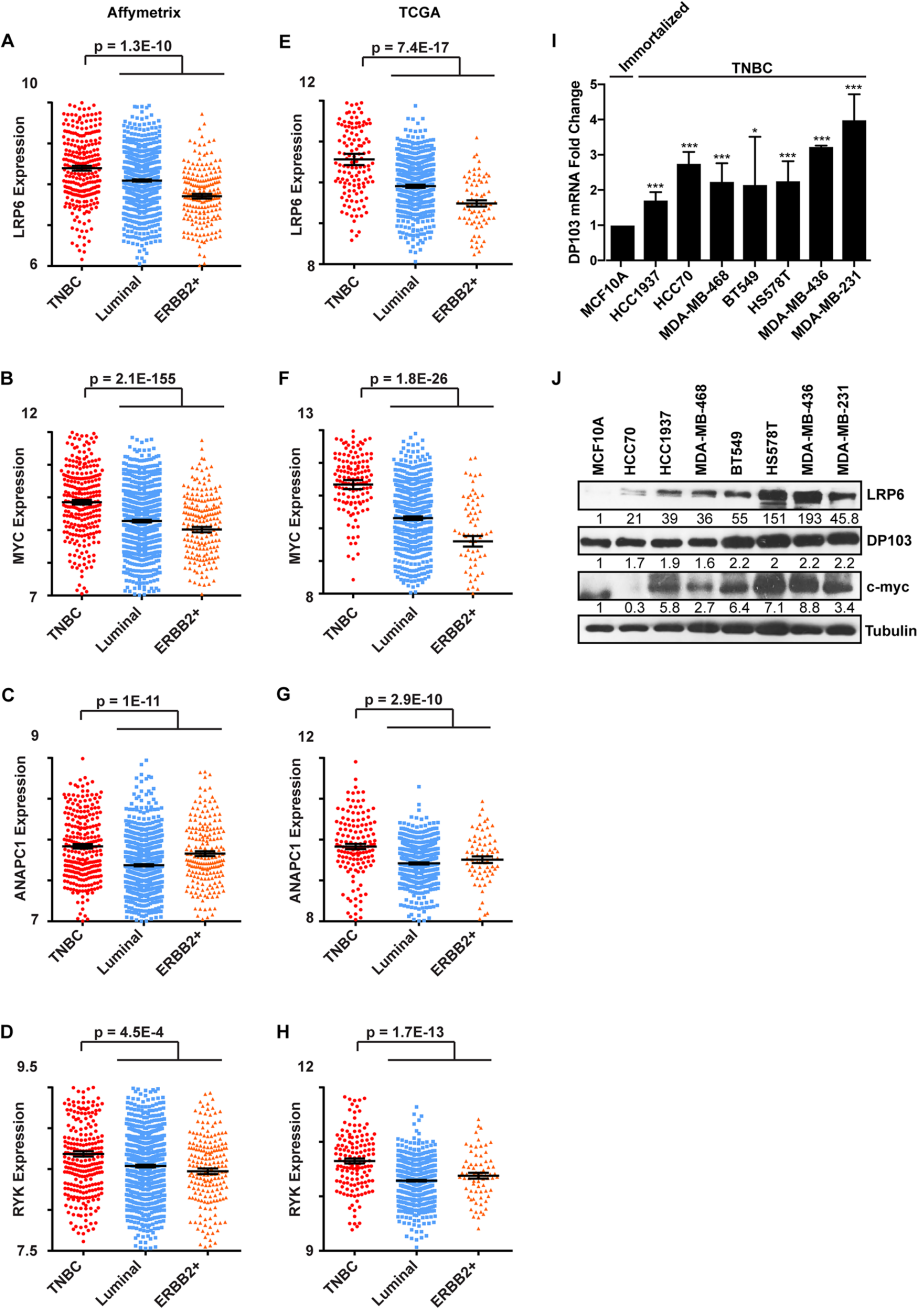
DP103 and Wnt proteins are highly expressed in TNBC. **A** LRP6 expression across different breast cancer subtypes in the Affymetrix cohort. **B** MYC expression across different breast cancer subtypes in the Affymetrix cohort. **C** ANAPC1 expression across different breast cancer subtypes in the Affymetrix cohort. **D** RYK expression across different breast cancer subtypes in the Affymetrix cohort. **E** LRP6 expression across different breast cancer subtypes in the TCGA cohort. **F** MYC expression across different breast cancer subtypes in the TCGA cohort. **G** ANAPC1 expression across different breast cancer subtypes in the TCGA cohort. **H** RYK expression across different breast cancer subtypes in the TCGA cohort. **I** qPCR analysis comparing mRNA expressions of DP103 across breast cell lines. Results represent the mean ± SD. *n* = 3. *p < 0.05, **p < 0.01, ***p < 0.001. **J** Cropped western blot analysis comparing protein expressions of DP103 and Wnt receptors LRP6, Wnt target gene c-Myc across breast cell lines.

We have previously reported that DP103 gene expression is aberrantly upregulated in breast cancer, in particular TNBC, compared to normal mammary tissue [32]. However, DP103 is rarely mutated or amplified/deleted in breast cancer (1.0% in 1492 breast cancer samples) (Fig. S1A), suggesting that DP103 expression is likely driven by factors other than mutation or copy number aberration. It has been shown that β-catenin mRNA levels are elevated in TNBC tumors compared to other subtypes [33]. Furthermore, increased stabilized nuclear or cytoplasmic β-catenin leads to increased Wnt/β-catenin activity, resulting in a poor prognosis in TNBC [8, 9, 34]. To determine if there is any correlation between breast cancer subtypes and aberrant Wnt signaling, we analyzed data from the Affymetrix meta-cohort and TCGA cohort. We stratified breast cancer into TNBC, luminal, and ERBB2^+^ and discovered that TNBC showed elevated Wnt modulators *LRP6*, *ANAPC1*, and *RYK*, as well as downstream target *MYC* gene expressions when compared against other subtypes in the Affymetrix cohort (Fig. 1A–D). Similar observations can be seen in the TCGA breast cancer cohort (Fig. 1E–H).

Consistent with our previous publication [32], upregulation of *DP103* mRNA was further confirmed by qPCR analysis (Fig. 1I) and protein levels by western blot analysis (Fig. 1J) in a panel of TNBC cell lines compared to the normal breast epithelial cell line (MCF10A) as a reference control. Interestingly, expression levels of LRP6, a key player in the Wnt/β-catenin signaling pathway, were observed to be significantly increased in the TNBC cell lines (Fig. 1J). Taken together, these results suggest that the Wnt signaling pathway may be active in TNBCs and positively correlates with high expression of DP103.

To determine if the correlation between DP103 and players of the Wnt pathway is conserved across different cancer types, we analyzed a gastric cancer (GC) patient-derived organoid RNAseq dataset. We observed a positive correlation (*r* = 0.49, *p* = 0.066) between expression of DP103 and Axin2 in these samples (Fig. S1B). To summarize the major biological themes between DP103-high and DP103-low samples, we performed enrichment analyses using the Hallmark, KEGG, Gene Ontology (GO), and Reactome databases. From these analyses, we highlighted a representative set of significantly enriched pathways related to Wnt signaling and associated regulatory processes identified across the different databases (Fig. S1C). Collectively, these results suggest an association between elevated DP103 expression and increased Wnt/β-catenin pathway activity in TNBC, with similar transcriptional relationships observed in an independent gastric cancer model.

### DP103 regulates Wnt pathway activity

**Fig. 2:**
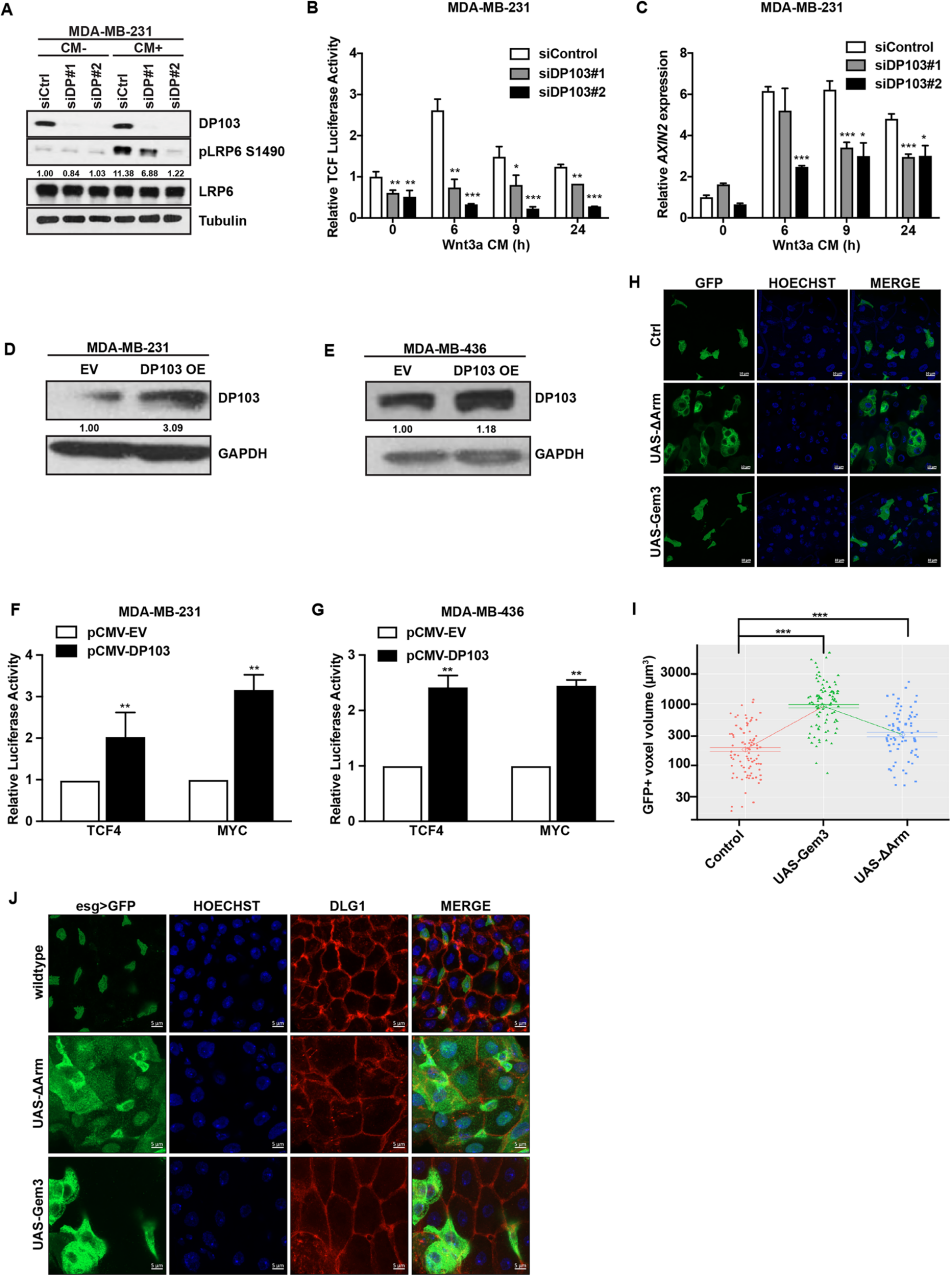
DP103 regulates the Wnt pathway through PTM of Wnt modulators. **A** Cropped western blot analysis comparing protein expressions of DP103 and Wnt receptors LRP6, Wnt protein Dvl2. **B** Effects of DP103 siRNA knockdown on TCF promoter activity with Wnt-3a CM stimulation in a time-dependent manner. Results shown represent mean relative TCF luciferase activity, measured by mean fold change of TOP/FOPflash luciferase units of DP103 siRNA against TOP/FOPflash luciferase units of control siRNA in each CM treatment time point ± SD, *n* = 3. *p < 0.05, **p < 0.01, ***p < 0.001. Significance is calculated against siControl. All luciferase readings are normalized with RenIa. **C** AXIN2 mRNA expression of siDP103 against siControl. Results represent the mean ± SD. *n* = 3. *p < 0.05, **p < 0.01, ***p < 0.001. Cropped western blot analysis showing overexpression of DP103 in MDA-MB-231 (**D**) and MDA-MB-436 (**E**). Effects of DP103 OE on TCF and MYC promoter activity in MDA-MB-231 (**F**) and MDA-MB-436 (**G**). Results shown represent mean relative TCF or MYC luciferase activity. Mean relative luciferase activity is measured by mean fold change of TOP/FOPflash luciferase units of treatment groups against TOP/FOPflash luciferase units of EV control ± SD, *n* = 3. *p < 0.05, **p < 0.01, ***p < 0.001. All luciferase readings are normalized with Renilla. **H** Expression of transgenic Wg/Wnt gain-of-function constructs in Drosophila intestinal stem cells. Images shown are representative of a minimum of three experiments. Volumetric quantification of GFP+ voxels of gemin3 and ΔArm guts in (**I**) using Imaris 9.0. ***p < 0.001. Five independent flies per condition were dissected for the quantification. Presented is the data from one representative experiment of three. **J** Gemin3 overexpression leads to mild loss of apico-basal polarity. Control esg>GFP midguts have their apico-basal polarity still intact, as shown through disks large 1 (dlg1) stain. Gemin3 mutants have a mild loss in apico-basal polarity as dlg1 is no longer tightly localized at the cell junctions. ΔArm mutants have a significant loss in polarity, whereby dlg1 is even more diffused as compared to Gemin3 and controls. Images shown are representative of a minimum of three experiments. See also Fig. S2.

To test whether DP103 regulates the Wnt/β-catenin pathway, protein expression of core Wnt pathway effectors was analyzed in TNBC cell lines MDA-MB-231 and MDA-MB-436 depleted of DP103 by siRNA. We confirmed Wnt pathway activation using Wnt3a-conditioned media (CM) or a GSK3 inhibitor (BIO). Both methods efficiently relocalized β-catenin into the nucleus (Fig. S1D and E). Treatment with Wnt3a-conditioned media resulted in phosphorylation of LRP6, confirming Wnt/β-catenin pathway activation (Figs. 2A and S1F). Interestingly, depletion of DP103 did not alter the total protein abundance of LRP6 but resulted in reduced levels of its phosphorylation (Figs. 2A and S1F). To confirm the effect of DP103 depletion on downstream signaling, we performed Wnt-responsive luciferase reporter assays on cells cultured in the presence or absence of Wnt3a-CM with or without DP103 depletion. Upon canonical Wnt pathway activation, we observed that depletion of DP103 reduced transcription of Wnt/TCF-driven luciferase (Fig. 2B) as well as the Wnt target genes, *AXIN2* (Fig. 2C). Conversely, overexpression of DP103 in two TNBC cell lines (Fig. 2D, E) resulted in increased transcriptional activity of TCF4 and downstream Wnt target, MYC (Fig. 2F, G). Taken together, the results indicate that DP103 modulates Wnt pathway activation.

In order to test overexpression of DP103 in vivo, we generated models with increased Wnt activity in *Drosophila* intestinal stem cells (ISC) by overexpression of a myristoylated, truncated version of armadillo (β-catenin homolog in Drosophila), which is tethered to the membrane and drives endogenous armadillo into the nucleus (UAS-ΔArm) [35, 36], and increased EGF signaling by overexpression of oncogenic Ras^V1237^. Both transgenes are under UAS-Gal4/UAS control, allowing expression in tissues of interest [37]. Expression in ISCs was accomplished by using the Escargot-Gal4 driver line in combination with UAS-GFP to mark ISCs [38]. Both overexpression mutants led to a noticeable increase in gut diameter (Fig. S2B), as well as highly proliferative stem cells and transit amplifying cells as marked by the mitosis-marker phosphorylated histone H3 (PH3) (Fig. S2C, quantified in Fig. S2D). ISCs can divide symmetrically, making an additional stem cell or asymmetrically generating a transit amplifying cell or Enteroblast (EB, Fig. S2A). EBs are proliferative and marked by a larger size and decreasing GFP corresponding to declining levels of the stem cell marker Esg. We observed the presence of larger cells marked by lower GFP levels (Fig. S2C), suggesting an increase in EB numbers and explaining the increase in PH3.

Overexpression of Gemin3 (DP103 homolog in *Drosophila*) (UAS-Gem3) led to a mild hyperproliferation phenotype, as observed through additional EBs (Figs. 2H, I and S2C, expression levels quantified in Fig. S2E). A previous report had shown that loss-of-function *Apc* mutant fly guts showed a loss of polarity, as visualized by the basolateral protein, Disks Large (Dlg1). Dlg1 is ordinarily restricted to just the junctions between enterocytes, becoming diffusely cytoplasmic [39]. We observed similar phenotypes with Gem3 and ΔArm mutants, where Gem3 displayed a mild loss of polarity phenotype with some cytoplasmic diffusion of Dlg1, while the ΔArm mutant was more severe with greater cytoplasmic diffusion of Dlg1 (Fig. 2J). Overall, the effect of strong Wnt activation using a gain-of-function β-catenin mimicked the previous findings for Apc mutants, while the phenotype of Gem3 (simple overexpression of the wildtype allele, rather than a gain-of- function allele) was less robust but did suggest that Gemin3 plays a role in controlling proliferation.

### DP103 interacts with GSK3β

**Fig. 3:**
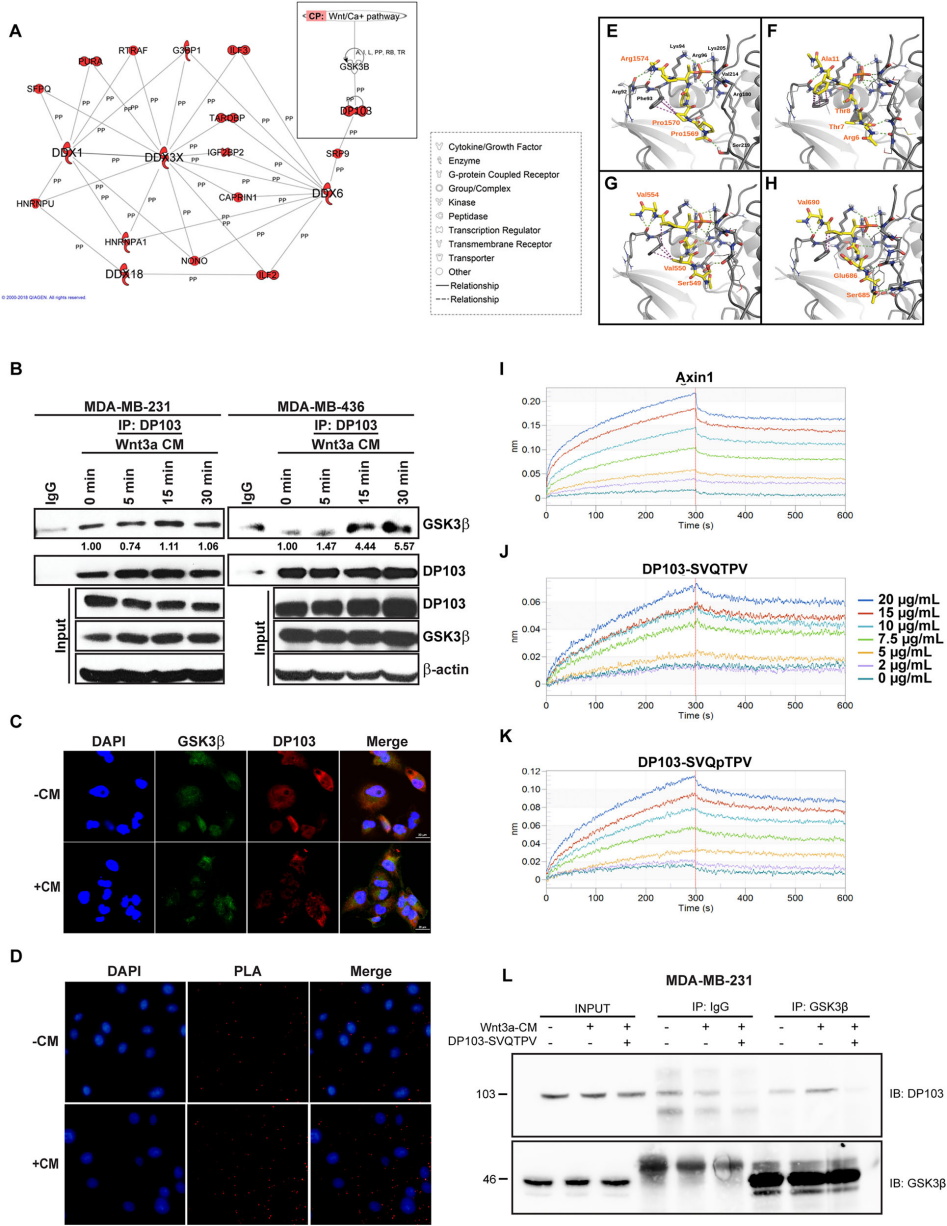
DP103 interacts with GSK3β. **A** IPA Network analysis showing protein-protein interactions of DDX super complex members. DP103 has direct protein-protein interaction with GSK3β, a member of the WNT pathway, and DDX1 and DDX3X proteins have direct protein-protein interaction with SYNCRIP (the receptor protein of RX-5902). The shape of the molecule represents the functional class of the gene product, as indicated in the legend. The red color indicates the proteins have H/L normalized ratio ≥ 1.3 in our SILAC data. **B** Co-immunoprecipitation of DP103 and GSK3β with Wnt-3a CM stimulation in TNBC cells, MDA-MB-231 and MDA-MB-436. **C** Immunofluorescence of DP103 (red) and GSK3β (green) in MDA-MB-231 cells. treated with 100 ng/mL of Wnt-3a recombinant protein for 15 min. **D** Proximal Ligation Assay analysis of DP103 and GSK3β in MDA-MB-231 TNBC cells. Phosphopeptide (yellow carbons; orange labels) binding to GSK3β (gray carbons; black labels) predicted modeling. **E** Phosphorylated LRP6 c-motif (PPP-pT-PR). **F** GSK3β autoinhibitory motif (RTT-pS-FA). **G** DP103 residues 549-554 (SVQ-pT-PV). **H** DP103 residues 685-690 (SES-pT-PV). Hydrogen bonding interactions shown as green dashes. Non-polar interactions are shown as purple dashes. Sidechains of GSK3β residues involved only in backbone-mediated hydrogen bonds are shown as lines. **I**–**K** BLI graphs depicting the real-time binding of a biotinylated peptide to GSK3β protein (0, 2, 5, 7.5, 10, 15, and 20 μg/mL). The red dotted line indicates the start of dissociation. The real-time binding curves were used to compute the equilibrium dissociation constant by nonlinear regression fitting using a 1:1 binding mode to the data. See also Fig. S3 and Tables S2–S4. **L** Pulldown of GSK3β and incubation with the non-phosphorylated 19-aa DP103-derived peptide (aa 542–560). Representative blot of two independent experiments.

To identify interactors of DP103, we utilized SILAC, which identified 339 proteins that have an H/L normalized ratio ≥ 1.3 (Table S1). These proteins were subjected to Ingenuity Pathway Analysis (IPA) to identify statistically significant canonical pathways and molecular networks. IPA predicted that DP103 may have a direct protein–protein interaction with GSK3β (Fig. 3A).

We confirmed this interaction via co-immunoprecipitation. Indeed, GSK3β could be detected following pull-down of DP103 in two cell lines, MDA-MB-231 and MDA-MB-436 (Fig. 3B). Furthermore, DP103 and GSK3β were also found to co-localize in the cytoplasm by immunofluorescence (Fig. 3C). This interaction was further confirmed using a proximal ligation assay (PLA) (Fig. 3D).

We then carried out molecular modeling to predict the region where interaction between GSK3β and DP103 occurred. Sixteen DP103-derived hexapeptides, encompassing known phosphosites in DP103, were investigated for binding to GSK3β (Table S2); these were the focus, as it is well known that GSK3β binds primed (i.e., prior phosphorylated) [40]. Binding energy calculations on the best-scoring docked positions of each peptide indicated that one DP103-derived phosphopeptide (SVQ-pT-PV; residues 549–554 of DP103) bound to GSK3β with significantly more favorable binding energy compared to the remaining DP103-derived phosphopeptides (Table S3). Furthermore, this peptide exhibited a significantly more favorable binding energy compared to both the LRP6- and GSK3β-derived peptides examined. The docked poses (Fig. 3E–H) of this DP103-derived peptide, the GSK3β autoinhibitory peptide, the LRP6 c-motif, and the DP103-derived SES-pT-PV peptide—with which the top-scoring DP103-derived peptide shares an identical sequence for four out of the six amino acids—were subject to molecular dynamics simulations followed by binding energy calculations (Fig. S3A–D, Table S4). These calculations further substantiated the binding strength of the DP103-derived SVQ-pT-PV peptide relative to the other peptides examined. Per-residue decomposition of the binding energies of the four peptides reveals that each of the six residues, the DP103-derived SVQ-pT-PV, contributes at least 3.0 kcal/mol to the binding energy with GSK3β, indicative of all six residues forming energetically favorable intermolecular interactions (i.e., negative binding energy) with GSK3β (Fig. S3E). By contrast, more variable individual contributions are made by residues in the other peptides, with some residues contributing unfavorably (i.e., positive binding energy) to the interaction. Collectively, these data suggest the high affinity of the DP103-derived SVQ-pT-PV for GSK3β.

To determine the strength of the binding between the DP103-derived phosphopeptide and GSK3β, biolayer interferometry (BLI) was carried out in the kinetic mode. GSK3β binding was quantified using streptavidin sensors loaded with biotinylated 19-aa-long peptides of either Axin1 (aa 383–401) or DP103 (aa 542–560). Purified full-length GSK3β protein bound strongly to the positive control peptide Axin1 with a *K*_d_ = 13.7 nM (*R*^2^ = 0.97). GSK3β protein bound DP103 peptide with a *K*_d_ = 34.5 nM (*R*^2^ = 0.98) and phosphorylated DP103 at threonine residue 552 with a *K*_d_ = 33.3 nM (*R*^2^ = 0.98) (Fig. 3I–K). Overall, the results indicate that GSK3β binds to DP103, although phosphorylation of T552 on DP103 did not affect the binding affinity significantly.

Consistent with the BLI findings, co-immunoprecipitation of GSK3β with incubation of the non-phosphorylated 19-aa DP103-derived peptide (aa 542–560) indicates that the peptide can outcompete binding and results in the dissociation of endogenous DP103 with GSK3β (Fig. 3L).

### DP103 is required for CSC formation, a downstream effect of the Wnt/β-catenin pathway

**Fig. 4:**
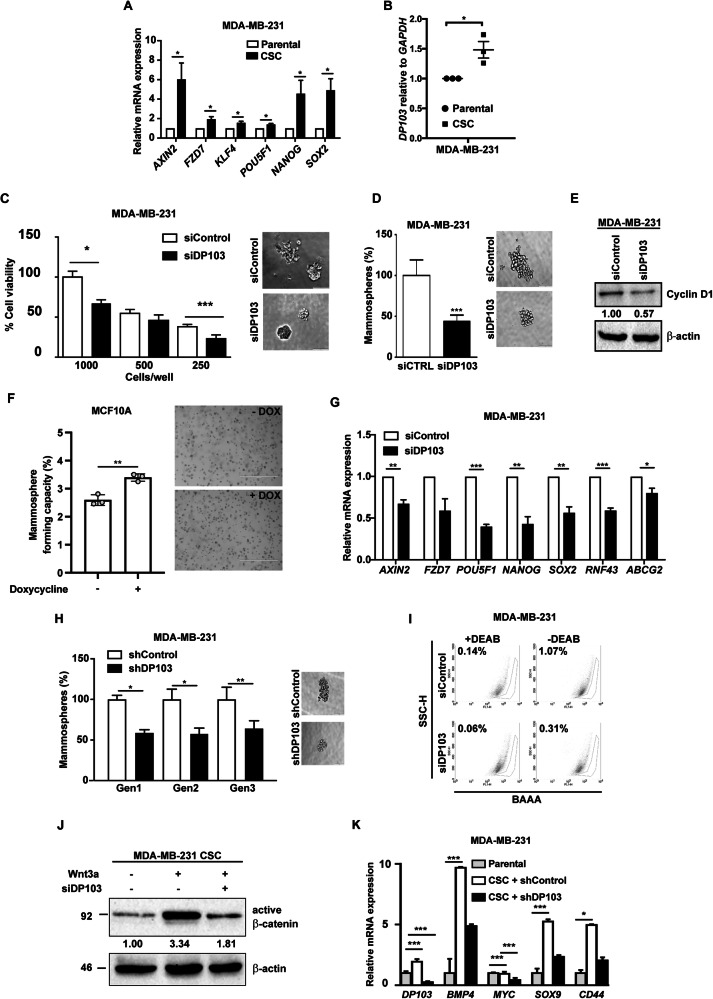
DP103 is required for CSC formation, a downstream effect of the Wnt/β-catenin pathway. **A** Gene expression of MDA-MB-231 monolayer parental and CSCs of *AXIN2, FZD7, KLF4, POU5F1, NANOG*, and *SOX2*. Values were calculated by the 2^−ΔΔCT^ method relative to *GAPDH*. Results represent the mean ± SD. *N* = 3. *p < 0.05. **B** Gene expression of *DP103* in MDA-MB-231 CSCs relative to parental cell lines. Values were calculated by the 2^−ΔΔCT^ method relative to *GAPDH*. *N* = 3. *p < 0.05. **C** MDA-MB-231 control and DP103 siRNA knockdown cells were re-seeded in triplicate in 3D Matrigel, serially diluted into 1000, 500, and 250 cells per 96-well. Scale bar of the representative pictures shown is 100 µm. Quantification of percentage cell growth was carried out using alamarBlue cell viability assay, where the absorbance of DP103 siRNA knockdown cells was compared against control cells, normalized to 100% ± SD, *n* = 3. *p < 0.05, ***p < 0.001. **D** Percentage mammosphere formation in MDA-MB-231 CSCs transfected with control siRNA or siRNA against DP103. Results represent the mean ± SD. *N* = 3. ***p < 0.001. **E** Cropped western blot analysis of MDA-MB-231 CSCs transfected with control siRNA or siRNA against DP103. Representative blot of two independent experiments. **F** Percentage mammosphere forming capacity in MCF10A cells, where overexpression of DP103 is induced by the addition of doxycycline. Results represent the mean ± SD. *N* = 3. **p < 0.01. **G** Gene expression of MDA-MB-231 CSCs 48 h after transfection with either control siRNA or siRNA directed at DP103 of *AXIN2, FZD7, POU5F1, NANOG, SOX2, RNF43*, and *ABCG2*. Values were calculated by the 2^−ΔΔCT^ method relative to *GAPDH*. Results represent the mean ± SD. *N* = 3. *p < 0.05, **p < 0.01, ***p < 0.001. **H** Percentage mammosphere formation in MDA-MB-231 CSCs infected with control or shRNA against DP103. Results represent the mean ± SD. *N* = 3. *p < 0.05, **p < 0.01. **I** ALDH1 stem cell marker expression in MDA-MB-231 control and DP103 siRNA knockdown TNBC cells was quantified using ALDEFLUOR Assay. Percentage of DEAB cells (negative control) was gated at 0%. Percentage of ALDH1-stained population of control and DP103 siRNA knockdown cells was normalized against their respective negative control DEAB cells to obtain the percentage ALDH1-positive cells. **J** Protein expression of active β-catenin by Western blotting of MDA-MB-231 CSCs after transfection of siDP103 with or without treatment with Wnt3a. Representative blot of three independent experiments. **K** qPCR results for gene expression of DP103 and CSC markers in cells obtained from monolayer parental adherent, control shRNA mammospheric, and shDP103 mammospheric MDA-MB-231 cells. Results represent the mean ± SD. *n* = 3. *p < 0.05, ***p < 0.001.

DP103 expression has been reported to be increased in induced pluripotent stem cells [41]. To determine if BCSCs exhibit increased DP103 expression like iPSCs, we enriched CSCs following published protocols [42–45]. using EGF/FGF/high-glucose media in suspension culture. We measured mRNA expression of Wnt target genes *AXIN2* and *FZD7*, and stem cell markers *KLF4, POUF51, NANOG,* and *SOX2*, which were all found to be upregulated compared to monolayer parental cells (Fig. 4A). Interestingly, when DP103 mRNA levels were assessed, we observed that CSCs had increased levels of DP103 mRNA (Fig. 4B).

To determine if DP103 regulates CSC genes, we performed an RNA-seq experiment and found that several CSC genes were upregulated while another set of CSC genes were downregulated upon DP103 depletion (Fig. S4A). Transiently depleted MDA-MB-231 and MDA-MB-436 cells of DP103 significantly decreased cell viability (Figs. 4C and S4B) and mammosphere growth in both cell lines compared to control cells (Figs. 4D and S4C). This coincided with a reduction in cyclin D1 as shown by western blot analysis (Fig. 4E). In addition, doxycycline-induced overexpression of DP103 in MCF10A resulted in an increase in mammosphere-forming capacity (Fig. 4F). We then investigated the role of DP103 on cell cycle progression of CSCs. Knockdown of DP103 in MDA-MB-231-derived CSCs significantly reduced S to G_2_ phase transition, while significantly increasing the percentage of cells in sub-G_0_ (Fig. S4D and E). Furthermore, gene set enrichment analysis (GSEA) on the TNBC subset of the METABRIC cohort revealed significantly enriched gene sets involved in cell cycle regulation in DDX20^high^ patients (Fig. S4F). To validate that DP103 could regulate Wnt signaling and stemness characteristics, we assessed mRNA levels of stem cell markers and Wnt target genes such as *NANOG*, *SOX2*, *POU5F1*, *ABCG2*, *AXIN2*, and *RNF43*, all of which were significantly reduced upon DP103 depletion (Fig. 4G).

To rule out the possibility that a decrease in mammospheres was not due to aggregation of quiescent cells, we examined the ability of primary mammospheres to form secondary and tertiary mammospheres upon stable integration of shDP103, showing reduced DP103 expression (Fig. S4G and H). We observed that MDA-MB-231-shDP103 cells exhibited an overall decrease in mammosphere growth in subsequent generations (Fig. 4H). Increased ALDH1 activity has been reported to identify CSC-like cell populations in breast cancer [46]. To test this, MDA-MB-231 and MDA-MB-436 cells depleted of DP103, and RNAi-control cells were subjected to ALDEFLUOR assay, which demonstrated a reduction in the ALDH1-positive cells in both cell lines after DP103 depletion (Figs. 4I and S4I). DP103 also regulated Wnt signaling in BCSCs, which was demonstrated by the reduction in active β-catenin in cells depleted of DP103 in the presence of Wnt3a (Fig. 4J), indicating DP103 is essential for Wnt activation. We also found a reduction in the gene expression of Wnt target genes responsible for stem cell formation, *BMP4*, *MYC*, *SOX9*, and *CD44* (Fig. 4K) [47], in mammospheres expressing shDP103, when compared to control monolayer parental cells, cultured in mammosphere media.

To assess the effects of DP103 on tumor initiation in vivo, we orthotopically injected MDA-MB-231 cells with or without shDP103 into mice. We found a significant decrease in the relative tumor size and metastasis to the lungs between scramble and DP103-depleted tumors at day 30 (*p* = 0.0386) (Fig. S5). These results demonstrate that the depletion of DP103 results in the suppression of tumor growth and metastasis.

### DP103 expression is regulated by the Wnt pathway

Many oncogenic signaling pathways are regulated by positive feedback loops; aberrant expression of proteins regulating an oncogenic pathway may also be transcriptional targets of the same oncogenic signaling pathway. To investigate if DP103 is also a Wnt target gene, TCF4 binding at the DP103 promoter was analyzed in the ENCODE ChIP-seq database. Interestingly, TCF4 binding peaks were observed at the promoter of the *DP103* gene in multiple cell lines, coinciding with the H3K27Ac chromatin signature, suggesting an active gene regulatory region (Fig. S6A). An increase in H3K27Ac at the DP103 locus in three TNBC cell lines indicates active transcription (Fig. S6B).

**Fig. 5:**
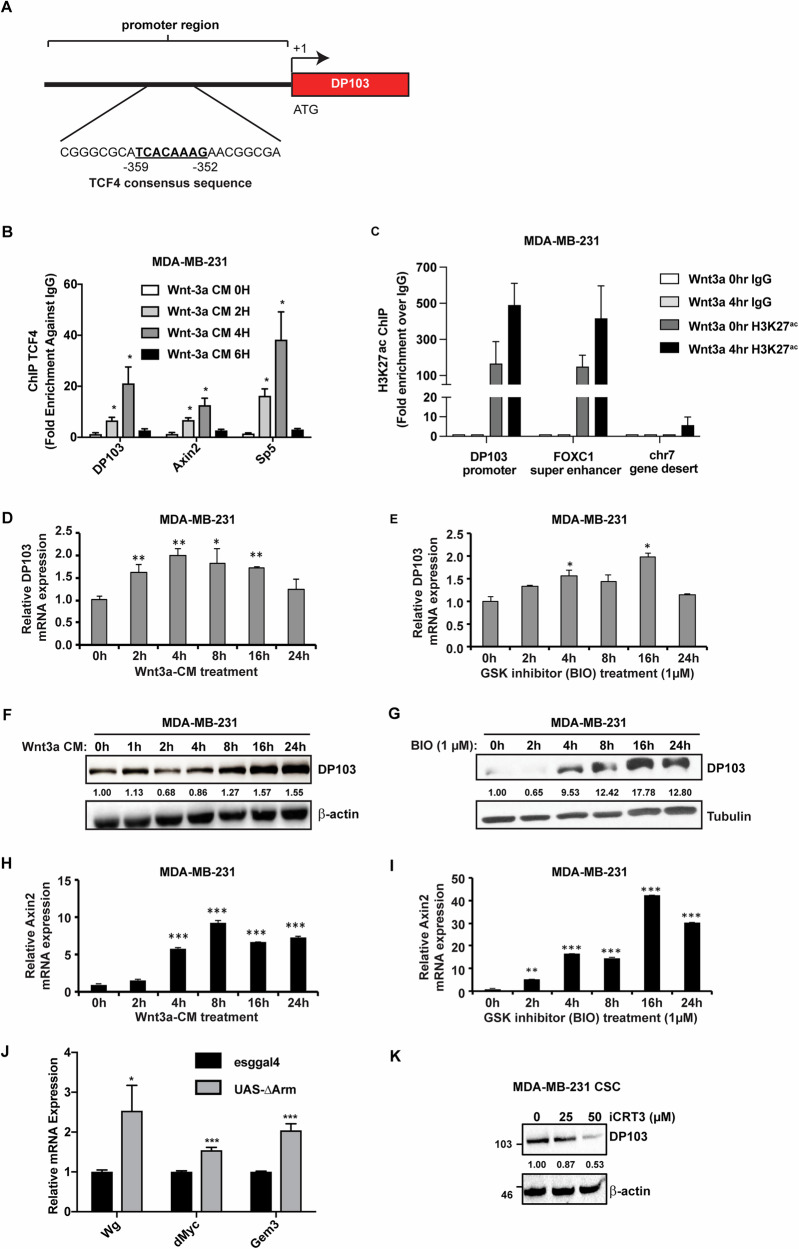
DP103 expression is regulated by the Wnt pathway. **A** Schematic showing the TCF consensus sequence at the promoter region of DP103. **B** ChIP TCF4 qPCR results for DP103 gene and known Wnt target genes Axin2 and Sp5 (positive controls) at various Wnt-3a CM treatment time points. Results shown represent mean Fold enrichment (ΔΔCt) ± SD, *n* = 3. *p < 0.05. **C** Chromatin immunoprecipitation of H3K27Ac was performed on MDA-MB-231 cells treated ± Wnt3a-CM for 4 h. Results represent the mean ± SD. *n* = 3. **D,** **E** Effects of Wnt/β-catenin stimulation by Wnt-3a CM and GSK3α/β inhibitor (BIO) on DP103 mRNA in MDA-MB-231. Results represent the mean ± SD. *n* = 3. *p < 0.05, **p < 0.01. **F,** **G** Effects of Wnt/β-catenin stimulation by Wnt-3a CM and GSK3α/β inhibitor (BIO) on DP103 protein expression in MDA-MB-231. **H**, **I** Effects of Wnt/β-catenin stimulation by Wnt-3a CM and GSK3α/β inhibitor (BIO) on *AXIN2* transcription in MDA-MB-231. Results represent the mean ± SD. *n* = 3. *p < 0.05, **p < 0.01, ***p < 0.001. **J** qPCR analysis on Wg, dMyc, and Gem3 mRNA in esggal4 and UAS-ΔArm Drosophila cells. Results represent the mean ± SD. *n* = 3. *p < 0.05, ***p < 0.001. **K** Cropped protein expression of DP103 by Western blotting of MDA-MB-231 CSCs 24 h after treatment with 0, 25, 50 µM iCRT (TCF4-/β-catenin inhibitor). All numbers represent the mean of at least three biological repeats normalized to β-actin.

Sequence analysis of the *DP103* promoter revealed TCF4 binding consensus [48], upstream to the *DP103* gene transcriptional start site (Fig. 5A). To validate this finding, ChIP qPCR was performed at various time points after stimulation with Wnt3a. A significant enrichment of TCF4 binding to DP103 promoter was observed at 2 and 4 h of Wnt-3a CM treatment which was significantly reduced at 6 h (Fig. 5B). This was mirrored in TCF-4 ChIP of Axin2 and Sp5 promoters, which served as positive controls (Fig. 5B). After 4 h of Wnt3a treatment, increased levels of H3K27Ac were found on the DP103 promoter (overlapping with TCF4 deposition—same primers used) compared to untreated control, indicating increased levels of transcription activation by Wnt driven TCF4-mediated transcription (Fig. 5C). Increased levels of H3K27Ac were found relative to IgG controls, when H3K27Ac was pulled down using primers targeting human FOXC1 super enhancer (positive control), and non-significant enrichment was found using primers targeting a gene desert located on chromosome 7 (Fig. 5C). This Wnt3a dependent binding of TCF4 on the DP103 promoter indicates that DP103 is a target of canonical Wnt signaling and mediates a positive feedback loop.

To confirm our observation that DP103 is a target of Wnt signaling we stimulated MDA-MB-231 and MDA-MB436 cells with Wnt-3a CM or a GSK3 inhibitor (BIO). We observed an increase in *DP103* mRNA (Figs. 5D, E and S6C–E) and protein levels (Figs. 5F, G and S6E, F) upon Wnt pathway activation in a time-dependent manner, which was mirrored in *AXIN2* mRNA expression (Figs. 5H, I and S6G, H). We confirmed this in vivo in *Drosophila*, where overexpression of β-catenin (UAS-ΔArm) also led to increased expression of ISC targets Wnt (Wg), dMyc, and DP103 (Gemin3) (Fig. 5J) [46]. To further confirm if DP103 transcription was dependent on β-catenin, we treated MDA-MB-231 CSCs with an inhibitor against β-catenin-responsive transcription (iCRT3) and observed a decrease in DP103 protein levels (Fig. 5K), indicating that DP103 was regulated by β-catenin in the Wnt pathway.

### RX-5902 decreases DP103 levels and reduces the survival of TNBC

**Fig. 6:**
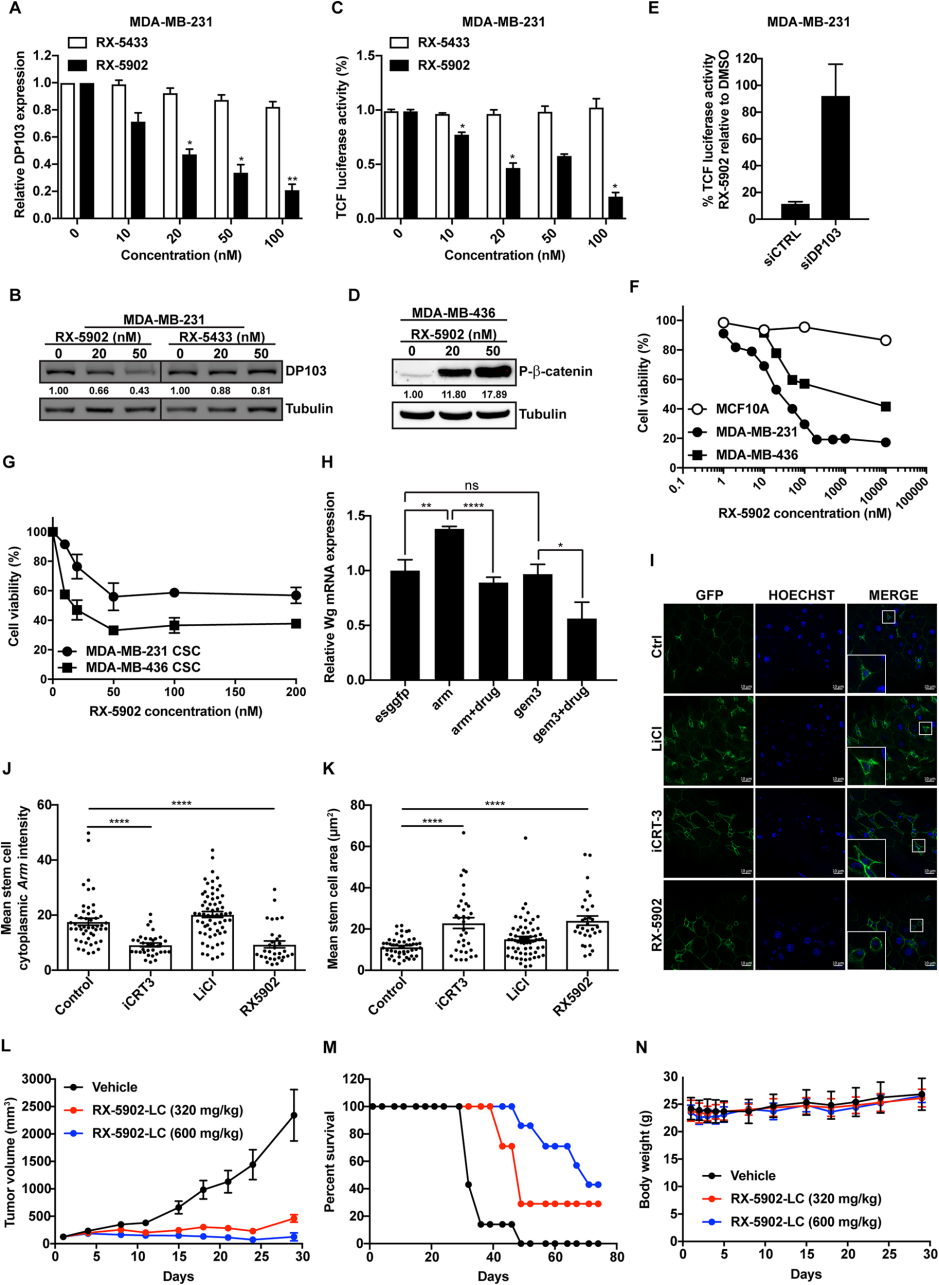
RX-5902 decreases DP103 levels and reduces the survival of TNBC cells. **A** Gene expression of DP103 in MDA-MB-231 cells treated with either RX-5902 or RX-5433 for 18 h. Values were calculated by the 2^−ΔΔCT^ method relative to *GAPDH*. Results represent the mean ± SD. *n* = 3. *p < 0.05, **p < 0.01. **B** Cropped protein expression of DP103 in MDA-MB-231 cells treated with either RX-5902 or RX-5433. Tubulin was used as a loading control. **C** TCF luciferase activity in MDA-MB-231 cells treated with either RX-5902 or RX-5433. Results represent the mean ± SD. *n* = 3. *p < 0.05. **D** Cropped western blot analysis of inactive P-β-catenin levels upon RX-5902 treatment in MDA-MB-436. **E** TCF luciferase activity in MDA-MB-231 cells treated with RX-5902 relative to DMSO. Results represent the mean ± SD. *n* = 3. **F** Cell viability of MCF10A, MDA-MB-231, and MDA-MB-436 cells treated with RX-5902. **G** Cell viability of MDA-MB-231 and MDA-MB-436 CSCs treated with RX-5902. **H** Treatment of RX-5902 (5 mM) in both UAS-Gemin3 and UAS-ΔArm shows a significant depletion in Wg mRNA. Results represent the mean ± SD. *n* = 3. *p < 0.05, **p < 0.01, ****p < 0.0001. **I** Effects of LiCl (5 mM), iCRT-3 (0.5 mM), and RX-5902 (5 mM) on armadillo localization visualized in adult midguts. Treatment with the GSK3 inhibitor LiCl results in an over-proliferation of stem cells and diffusing of armadillo from cell junctions, while treatment with Wnt inhibitors iCRT-3 and RX-5902 results in a decrease in stem cell count and tighter armadillo localization to cell junctions. The inset shows zoomed images. Images shown are representative of a minimum of three experiments. **J** Cytoplasmic Arm intensity was quantified by outlining the stem cells with high membranous arm intensity. Only the cytoplasmic and not the membrane Arm was quantified. Data presented is the pool of at least three repeats with a minimum of 9 stem cells quantified per image. One-way ANOVA was performed (*p* < 0.0001****), followed by unpaired *t*-tests. **K** Size of stem cells was calculated from the area of the cytoplasm measured for Arm intensity. Quantified area is presented as a pool of at least three repeats with a minimum of 9 stem cells quantified per image. One-way ANOVA was performed (*p* < 0.0001****), followed by unpaired *t*-tests. **L** Tumor volumes of tumor-bearing mice treated with either Vehicle or the indicated doses of RX-5902-LC. Results represent the mean ± SD. *n* = 3. **M** Survival plots of tumor-bearing mice treated with either Vehicle or the indicated doses of RX-5902-LC. Results represent the mean ± SD. n = 3. **N** Mean body weight of tumor-bearing mice treated with either Vehicle or the indicated doses of RX-5902-LC. Results represent the mean ± SD. *n* = 3.

Phosphorylation on tyrosine residue 593 on DDX5 has been shown to bind to β-catenin and promote its translocation into the nucleus [49, 50]. A novel first-in-class, orally bioavailable Wnt inhibitor, RX-5902 (Rexahn Pharmaceuticals, Inc., USA), was recently discovered to inhibit phosphorylated DDX5 and β-catenin nuclear translocation, thereby reducing canonical Wnt-induced signaling and downstream Wnt-associated genes transcription, demonstrating anti-tumor properties [51]. Given that DP103 is an oncogene and that its transcription is dependent on β-catenin, we sought to investigate if we could reduce the levels of DP103 by inhibiting nuclear β-catenin localization using DDX5 inhibitor RX-5902. We tested the effects of RX-5902 on MDA-MB-231 and MDA-MB-436 cells. Treatment with RX-5902, but not its inactive analog RX-5433, significantly reduced DP103 mRNA (Figs. 6A and S6A) and protein expression (Figs. 6B and S6B) in a dose-dependent manner. Treatment with RX-5902 was shown to reduce Wnt/β-catenin activity (Figs. 6C and S6C) and increase inactive P-β-catenin levels (Fig. 6D). Furthermore, we found that this reduction in Wnt/β-catenin activity is abolished when DP103 is depleted (Figs. 6E and S6D), suggesting that the effects of RX-5902 are partly dependent on DP103. We then treated MDA-MB-231 and MDA-MB-436 with RX-5902 and measured cell viability. We compared these cell lines with the normal breast epithelial cell line MCF10A. RX-5902 treatment specifically reduced the cell viability of TNBC cell lines with minimal impact on the MCF10A cell line (Fig. 6F). Furthermore, treatment of RX-5902 could reduce the viability of MDA-MB-231 and MDA-MB-436 CSCs (Fig. 6G).

To test if RX-5902 works in vivo, we fed *Drosophila* with varying doses of RX-5902 and measured *wg* transcript levels. *Drosophila* fed with 5 mM of RX-5902 showed a significant decrease in *wg* transcripts (Fig. S7E). A similar decrease in *wg* mRNA expression levels was observed upon treating high-Wnt Δarm mutants and Gemin3 mutants with RX-5902 (Fig. 6H). Interestingly, *wg* mRNA drops below basal levels in Gemin3 mutants, which suggests that the drug is effective in inhibiting Wnt activity in these flies. Notably, flies tolerated RX-5902 treatment for several days without lethality, indicating that the compound is not acutely toxic in this model.

To better visualize the effect of RX-5902, we utilized an Arm-mimic-GFP fly that has an eGFP cassette inserted into the *armadillo* gene at its endogenous locus. It can thus produce cellular outline images of the fly gut due to the β-catenin localized at the cell junctions and show Wnt-responding cells by increased cytoplasmic and nuclear GFP signal. Using the arm-mimic-GFP fly, we imaged intestinal stem cells as well as the enteroblasts and enterocytes (differentiated cells) (Fig. 6I, GFP panels). The stem cells are noticeably smaller in size and have very small nuclei, whereas the enterocytes are much larger in size and possess much larger nuclei due to endoreplication of DNA (Fig. 6I, Schema in Fig. S2A) [52]. Intestinal stem cells possess greater GFP intensity, indicating higher levels of armadillo diffused in the cell due to Wnt signals from niche cells [53]. Treatment with GSK3β inhibitor lithium chloride (LiCl) led to an increase in stem cell number as well as greater Arm diffusion inside the cells (Fig. 6I–K). Flies treated with iCRT-3 led to a significant decrease in stem cell count, and Arm was more tightly localized within junctions and not diffused in cells (Fig. 6I–K). Treatment with RX-5902-LC (lyophilized cake) led to a similar Wnt-inactivating effect comparable to that of iCRT-3 (Fig. 6I–K). These results indicated that RX-5902 was able to inhibit the Wnt pathway activity in vivo.

To determine if RX-5902 is effective against mouse intestinal organoids, intestinal organoids of different genotypes: wild-type, Apc−/− (Wnt activated), and KPN (high metastatic potential) were treated with RX-5902. There was a significant difference between the wild-type and Apc and/or KPN organoids at 50 and 100 nM, indicating that this would be a therapeutic window where the majority of normal intestinal epithelium would remain viable (Fig. S7F and G). Importantly, RX-5902 treatment in MDA-MB-231 tumor-bearing mice resulted in a reduction of tumor volume and extended the survival of mice without much effect on body weight (Fig. 6L–N). Taken together, the results indicate that RX-5902 could represent an effective drug for targeted therapy.

Building on these observations, we next evaluated the potential of RX-5902 to enhance therapeutic efficacy when combined with existing chemotherapy and targeted therapies used for TNBC. In three-dimensional MDA-MB-231 tumor spheroids, co-treatment with RX-5902 and poly ADP ribose polymerase (PARP) inhibitor, olaparib, resulted in greater reduction in cell viability compared with either agent alone (Fig. S7H). These findings suggest that RX-5902 may synergize with PARP inhibition in this model and warrant further investigation of this combination strategy in TNBC.

## Discussion

Our data have revealed a role for DEAD-box RNA helicase DP103 in the regulation of the Wnt/β-catenin signaling pathway in TNBC, independent of its canonical RNA helicase domain and presumably function. It was previously demonstrated that DP103 was overexpressed in TNBC and resulted in increased metastatic disease through a TAK1-NF-κB regulatory mechanism, although our data suggest DP103 regulates multiple signaling pathways [32]. The Wnt/β-catenin signaling pathway is commonly hyperactivated in TNBC, and may be the result of overexpression of β-catenin and LRP6 [7]. We also found that DP103 positively correlated with the mRNA expression of Wnt targets *LRP6* and *MYC*, and high DP103-expressing TNBC cell lines had increased levels of LRP6 phosphorylation, a PTM required for sustained Wnt activation through docking of multiple receptors, forming ‘signalosomes’ [54, 55]. The depletion of DP103 decreased phosphorylation of LRP6 and nuclear β-catenin levels. Furthermore, the overexpression or depletion of DP103 resulted in increased or decreased Wnt-driven transcriptional activity, respectively. Our data demonstrated that DP103 modulates Wnt signaling by activating PTMs of various Wnt signaling components, LRP6 and β-catenin, which are both partially regulated through the kinase activity of GSK3β [56]. Notably, analysis of an independent gastric cancer patient-derived organoid RNA-seq dataset revealed a positive correlation between DP103 expression and the canonical Wnt target AXIN2, along with enrichment of Wnt-related pathways, suggesting that the association between DP103 and Wnt signaling may extend beyond TBC to other cancer contexts.

Given the importance of GSK3β in regulating the Wnt pathway, we have shown that the interaction between DP103 and GSK3β is likely the mechanism by which DP103 regulates Wnt. Previous studies have demonstrated that the RNA-binding and RNA-metabolism independent roles of DP103 primarily occur through protein–protein interactions in this region [26]. By activating Wnt signaling upstream of β-catenin, our data also confirmed that DP103 itself is a target of downstream TCF4-mediated transcription. This provides evidence of a positive feedback loop—common in oncogenic cell signaling [57, 58]. We demonstrated that Wnt3a stimulation resulted in an increase in DP103 expression. It is quite possible that in vivo, increased DP103 expression is driven by enhanced paracrine Wnt3a signaling from stromal tissues, a key driver of breast cancer oncogenesis [59]. This would further reinforce DP103-driven Wnt signaling in TNBC and breast cancer progression.

Breast cancer progression can partly be attributed to a population of cells, known as CSCs, which exhibit characteristics that promote drug resistance and metastasis [60]. The involvement of DP103 in promoting metastatic potential in breast cancer [32], led us to investigate whether DP103 could impact on promoting CSC-like cells in basal breast cancers. We have demonstrated that DP103 promotes CSC-like traits in two basal B breast cancer cell lines. DP103 mediates the self-renewal capacity of ALDH1^+^ and CD44^high^/CD24^low/-ve^ cell populations contributing to the stemness of BCSCs [58]. The stemness characteristics of these cells are driven by transcription factors Nanog, Oct4, and Sox2 [61–63]. These transcription factors are also transcriptional targets of Wnt signaling [64, 65]. The reduction in their gene expression evident after DP103 depletion is likely a consequence of the upstream effects on Wnt/β-catenin signaling, which DP103 promotes. We have further demonstrated a reduction in Wnt targets *BMP4*, *MYC*, *SOX9*, and *CD44,* which may also mediate signaling via the NF-kB pathway to promote metastasis and therapeutic resistance in many cancers [66].

The findings in Drosophila are not in complete concordance with the role of DP103 in human TNBC cell lines. We observed that Gemin3 overexpression leads to overproliferation and loss of polarity, consistent with the role of DP103 in driving tumor formation and epithelial-to-mesenchymal transition (EMT) [32]. However, the effects of overexpressing DP103 in TNBC were more severe compared to the Gemin3 overexpression model in *Drosophila*, which displayed a milder phenotype. These differences may be explained by the different approach in vivo, where only a small subset of cells (ISCs) in the intestinal epithelium expressed Gem3, or by the varied roles that Gemin3/DP103 plays depending on the physiological or pathological contexts. The overexpression of Gemin3 in an in vivo physiological model is not sufficient to produce oncogenic effects, whereas DP103 may regulate Wnt-driven tumorigenesis in vitro by cooperatively associating with other dysregulated and overexpressed components of the Wnt pathway. While the phenotypes between our two models differ, our data demonstrate the role of DP103 in regulating Wnt signaling in vitro and in vivo in both a physiological and pathophysiological context.

Clinically, TNBC presents with many characteristics that increase the difficulty of effective treatment. Limited successful molecular targets exist in TNBC; RX-5902, a first-in-class, orally bioavailable Wnt inhibitor currently in Phase I/IIa trials. Our pre-clinical data obtained from RX-5902 have shown promising results, as treatment of TNBC cells with RX-5902, but not the inactive analog RX-5433, showed a significant decrease in Wnt/β-catenin activity, which was abrogated upon DP103 depletion, demonstrating that DP103 is essential for RX-5902 efficacy. Furthermore, DP103 expression (mRNA and protein) was reduced with RX-5902 treatment, confirming that RX-5902 is a Wnt pathway-specific therapeutic and that DP103 is a target of the Wnt pathway. RX-5902 treatment also decreased cell viability of TNBC cells and mammospheres in a dose-dependent manner, although largely sparing the normal epithelial cell line MCF10A. In vivo, we show that RX-5902 is effective in reducing tumor volume and prolonging the survival of tumor-bearing mice. Notably, in three-dimensional TNBC tumor spheroids, RX-5902 co-treatment with the PARP inhibitor olaparib resulted in enhanced anti-tumor effects compared with either agent alone, suggesting that RX-5902 may synergize with existing targeted therapies.

While our study demonstrates that RX-5902 effectively reduces DP103 expression and suppresses Wnt signaling, available evidence to date predominantly addresses its therapeutic effects, and the long-term safety profile remains to be defined. Preclinical studies and early-phase clinical evaluations indicate that RX-5902 is generally well tolerated, with reported adverse events being predominantly low-grade and mainly consisting of gastrointestinal and constitutional symptoms such as nausea, vomiting, diarrhea, anorexia, fatigue, and weight loss [67, 68]. However, these findings originate from early-stage clinical studies with limited cohort size and follow-up duration. Given the essential roles of Wnt signaling and DP103-associated pathways in normal tissue homeostasis, further investigations incorporating extended dosing and systematic toxicity assessments will be important to fully establish the therapeutic window and translational potential of RX-5902. Collectively, these findings highlight the translational potential of RX-5902, both as a single agent and as part of rational combination strategies for the treatment of TNBC.

**Fig. 7:**
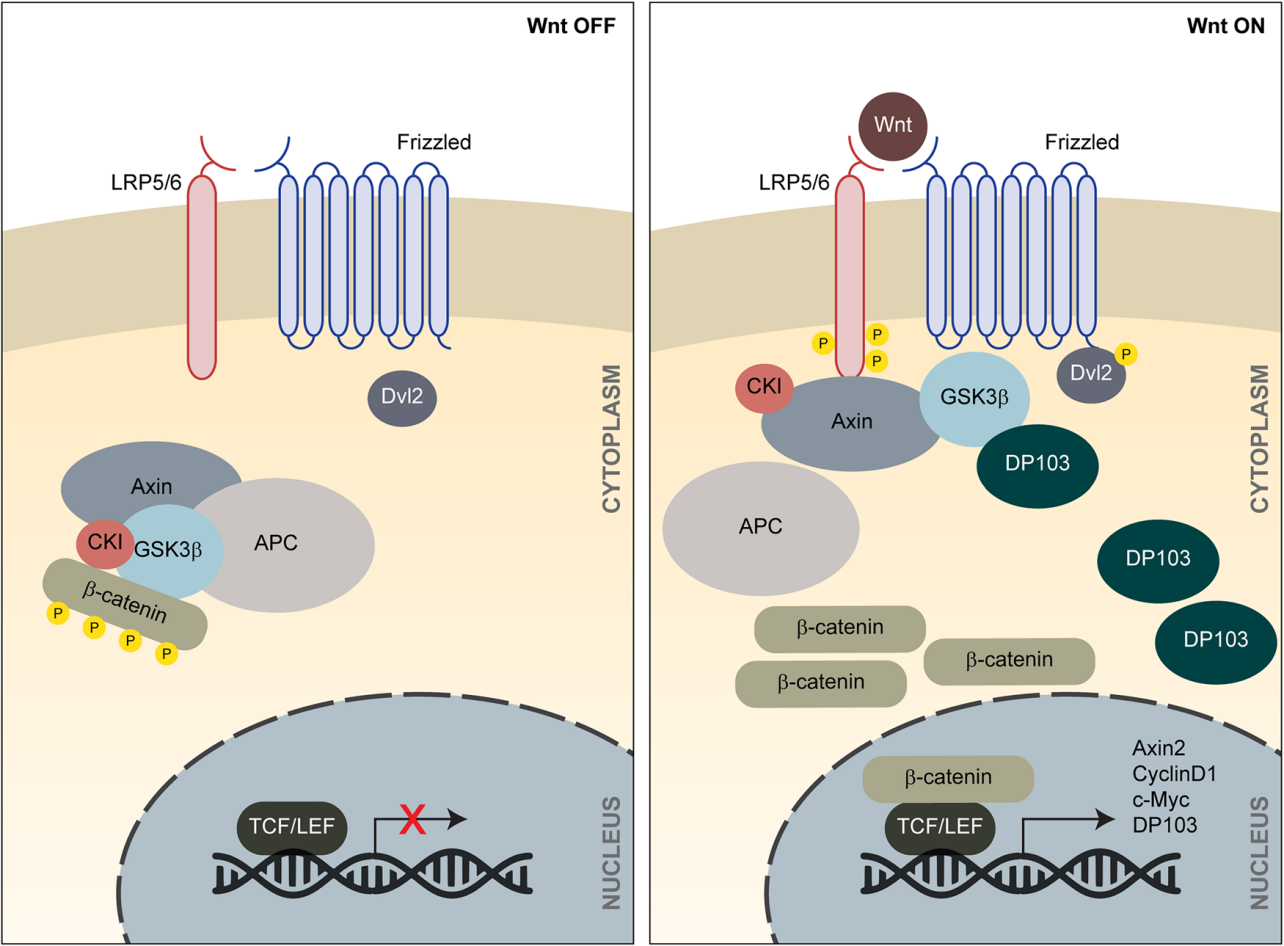
Schematic model. Under conditions where DP103 levels are low, phosphorylation in LRP5/6 and Disheveled is reduced, leading to minimal Wnt/β-catenin signaling and Wnt target genes transcription (including DP103), and overall reduction in cell proliferation, tumor initiation, and CSC properties in Wnt-responsive TNBCs. In TNBC cells, the presence of high levels of DP103 and its interaction with GSK3β leads to increased phosphorylation of LRP5/6 and Disheveled, positively modulating downstream Wnt activity and Wnt target genes transcription. DP103 itself as a Wnt target gene resulted in a positive feedback loop, further enhancing and sustaining the Wnt/β-catenin signaling pathway, leading to increased cell proliferation, tumor initiation, and CSC properties in Wnt-responsive TNBCs.

Taken together, our data identify a novel regulatory role of DP103 in the Wnt/β-catenin signaling pathway (Fig. 7) in both parental and CSC-derived TNBC cells. Furthermore, we show that DP103 can regulate Wnt signaling in a physiological context in vivo. Importantly, the concordant findings observed in three-dimensional TNBC tumor spheroids and human gastric cancer patient-derived organoids strengthen the translational relevance of our study and support the applicability of these mechanisms to human disease. Collectively, these results suggest that DP103 may present as a diagnostic biomarker and as an attractive target for breaking the positive feedback loop for sustained aberrant Wnt/β-catenin activity.

## Materials and methods

### Cell lines and culture conditions

All cell lines used in this study were obtained from American Type Culture Collection (ATCC, MD, USA). Cells lines were maintained in Dulbecco’s modified Eagle medium (DMEM) (Life Technology, USA) for tumor cell lines and mammary epithelial growth medium (MEGM) for normal epithelial cell lines supplemented with 10% FBS, 2 mM L-glutamine, Penicillin/Streptomycin 100 U/mL/100 µg/mL and a growth factor kit containing Bovine Pituitary Extract (BPE), hydrocortisone, hEGF, Insulin and gentamicin (Lonza, MD, USA) in a 37 °C incubator with 5% CO_2_. Epidermal growth factor (EGF), basic FGF (bFGF), bovine insulin, and B27 were purchased from Life Technologies, USA.

### Wnt-3a conditioned media preparation

Control and Wnt-3a conditioned media were prepared from L cells (ATCC CRL-2648) and L Wnt-3A cells (ATCC CRL-2647), respectively, through harvesting spent media from 80% confluent cells, after 72 h. Each batch of conditioned media was tested for the amount and activity of Wnt-3a protein by Western blot analysis and TOP/FOPflash luciferase reporter assay, respectively, to ensure a consistent dose of Wnt stimulation between batches. Wnt-3a recombinant protein (R&D Systems, MN, USA) was also used to validate experiments carried out using Wnt-3a conditioned media.

### Drosophila handling and fly stocks

Fly stocks were raised at 25 °C and kept in vials with standard fly food under constant light exposure. All crosses were performed at 25 °C. *esg* > *GFP* flies were used as control flies, unless stated otherwise. The following fly lines were used for intestinal stem cell tracking: (1) *esg* > *GFP* (*esg-Gal4, UAS-GFP)* [38], (2) *esg-Gal4, UAS-GFP/UAS-ΔArm*, (3) *esg-Gal4 UAS-GFP; UAS-Gem3*, (4) *esg-Gal4 UAS-GFP*; *UAS-Ras*^*V12*^, (5) *esg-Gal4 UAS-GFP*/*UAS-ΔArm*; *UAS-Ras*^*V12*^. The following fly line was used for live cell imaging and Arm levels: (6) Mi[PT-GFSTF.1]armMI08675-GFSTF.1 [69]. The loss-of-function Gem3^B^ and the overexpression UAS-Flag-Gemin3 fly stocks were gifts from Prof. A Gregory Matera, University of North Carolina, Chapel Hill, USA [70]. Two ubiquitous drivers were used in combination for the overexpression of UAS-Gem3, armadillo-GAL4, and daughterless-GAL4 [37]. The double GAL4 driver line and Oregon R flies were used as wild-type controls.

### Drug preparation and treatment in Drosophila

10 mL of fly food was heated in a hot water bath at 60 °C to generate a viscous consistency, whereby drugs can be homogenously added. Fly food was allowed to cool for 10 min to prevent drug decomposition due to high heat, followed by the addition of the drugs in appropriate molar concentrations. Drugs were mixed well into the food using a spatula and transferred into new vials, forming a thin layer (2–5 mm) of drug-infused fly food, which was left to air dry and re-solidify overnight. Food was then stored at 4 °C until use for experiments. Flies were fed with drug-infused food for 48 h, unless stated otherwise.

### Confocal microscopy

Whole gut images were obtained using LSM800 W Plan-Apochromat 10x, whereas close-ups of gut sections were done using W Plan-Apochromat ×63/1.4 Oil (Carl Zeiss). The LSM800 microscope was equipped with four diode laser lines: 5 mW 405 nm (blue—for DAPI/Hoechst stains), 10 mW 488 nm (green—for *esg*>*GFP* live imaging or AlexaFluor™ 488 stains), 10 mW 561 nm (red—for AlexaFluor™ 555 stains), and a 5 mW 640 nm (far-red—for AlexaFluor™ 647 stains). All images were taken with default scan speed and averaging of 4 with the recommended optimal z-stack steps. Maximum intensity projections and three-dimensional rendering were done using the ZEN Blue software [71].

### Live imaging of the adult fly gut

The *esg-Gal4, UAS-GFP*, and *armMI08675-GFSTF* lines were both used as live imaging models. *esg-Gal4, UAS-GFP* flies allowed the tracking and visualization of intestinal stem cells (ISCs) and enteroblasts (EBs), and can be further used to study the effects of transgenes of interest. The *armMI08675-GFSTF* [72] line, on the other hand, has eGFP inserted into *Armadillo* at its endogenous locus and provides a novel method of visualizing endogenous *Armadillo* localization. Here, we utilized the *armMI08675-GFSTF* [72] model and repurposed it to visualize enterocytes (EC) as well as effects of transgene expression and/or drug treatment on endogenous *Armadillo* localization patterns.

For live imaging of the fly midgut, samples were dissected in 200 µL of 1x PBS in a PYREX™ Spot Plates concave glass dish (Fisher Scientific). Hoechst 33342 nuclear stain (Thermo Fisher Scientific) running stock solution was prepared by diluting the stain 1:2000 in 1x PBS. 1 µL of the running stock was added to the gut samples and protected from light for 5 min. Subsequently, the guts were carefully transferred onto a small droplet of 1X PBS on a 35 mm glass-bottom dish. Using fine forceps, the gut was repositioned to resemble its natural orientation. PBS was then carefully removed from the area surrounding the gut, ensuring that the surface tension of the liquid holds the gut in place. Some excess PBS was left to prevent the gut tissue from undergoing desiccation while live imaging. The 3 mm glass-bottom dish was then mounted onto the LSM800 for imaging.

### Immunofluorescence histochemistry

Adult fly midguts were dissected in 1X PBS using the PYREX™ Spot Plates concave glass dish (Fisher Scientific). All subsequent steps, including antibody staining, were performed in the concave glass dish under a standard light microscope. Guts were fixed using a Fixation solution (4% paraformaldehyde in 1X PBS) for 45 min at room temperature and subsequently washed thrice using 1X Wash buffer (PBS, 0.1% Triton X-100). Blocking solution (PBS, 0.1% Tween-20, 10% BSA) was then added to the guts to prevent non-specific binding of antibodies for 1 h at room temperature, then subsequently washed thrice with 1X Wash buffer again. The samples were then incubated in primary antibodies of interest (antibodies were diluted in PBS, 0.5% Triton X-100) at 4 °C overnight. After thorough washing with 1X Wash buffer, samples were then incubated with secondary antibodies (also diluted in PBS, 0.5% Triton X-100) for 2 h at room temperature and kept in the dark. 30 min before the completion of the secondary antibody incubation, 1 µL of Hoechst 33342 nuclear stain running stock was added. Specific mounting setup of specimens was performed as described in ref. [73].

The primary antibodies used were chicken anti-GFP (Abcam, 1:10,000), mouse anti-GFP (Abcam, 1:500), mouse anti-Arm (Developmental Studies Hybridoma Bank (DSHB), N2 7A1, 1:20), mouse anti-Disks large 1 (DSHB, 4F3, 1:20), and rabbit anti-phospho-histone H3 (Ser10) (Millipore, 1:1000). Secondary antibodies used were Alexa Fluor™ 488, 555, and 647 Goat anti-Mouse IgG (Thermo Fisher, 1:500), Alexa Fluor™ 555 and 647 Goat anti-Rabbit IgG (Thermo Fisher, 1:500) and Alexa Fluor™ 488 Goat Anti-Chicken IgY (Thermo Fisher, 1:500). All images were taken using the LSM800 and processed in ZEN Blue.

### Quantification and statistics

Z-stack images taken from the LSM800 were transferred to the Bitplane Imaris 9.0 microscopy image analysis software (Bitplane) for analysis of overproliferative qualities. The first method was to locate GFP+ cells using the Volume protocol. Independent voxels of GFP+ cells within a ×63 field that overlapped with the blue Hoechst stain were counted as an individual cell. For phospho-histone H3 quantification, the same methodology was used but for PH3+ cells. The second method was a relative quantification of GFP intensity per gut section, which was performed by measuring overall GFP intensity for the gut section and normalized by the total number of cells in the field. Gut width and cell counting were done using ImageJ (NIH). All data were recorded, statistically analyzed, and graphed using Prism 6 (GraphPad Software) and RStudio.

### RX-5902 studies in mice

Female athymic nude mice (CRL: NU(NCr)-Foxn1nu, Charles River) were 10-week old with a body weight (BW) range of 16.8–28.1 g, on Day 1 of the study. The animals were fed ad libitum water (reverse osmosis, 1 ppm Cl), and NIH 31 Modified and Irradiated Lab Diet® consisting of 18.0% crude protein, 5.0% crude fat, and 5.0% crude fiber. The mice were housed on irradiated Enrich-o’cobs™ Laboratory Animal Bedding in static microisolators on a 12-h light cycle at 20–22 °C (68–72 °F) and 40–60% humidity. CR Discovery Services specifically complies with the recommendations of the *Guide for Care and Use of Laboratory Animals* with respect to restraint, husbandry, surgical procedures, feed and fluid regulation, and veterinary care. The animal care and use program at CR Discovery Services is accredited by the Association for Assessment and Accreditation of Laboratory Animal Care International (AAALAC), which assures compliance with accepted standards for the care and use of laboratory animals.

Tumors were initiated via subcutaneous (s.c.) injection of MDA-MB-231 cells into the right flank of female athymic nude mice. The cells were harvested during exponential growth and resuspended at a concentration of 5 × 10^7^ cells/mL in PBS. Each test animal received an s.c. injection of 5 × 10^6^ MDA-MB-231 cells (0.1 mL cell suspension) into the right flank, and tumor growth was monitored as the average tumor size approached the target range of 100–150 mm^3^. Tumors were measured in two dimensions using calipers, and volume was calculated using the formula:

$$Tumor\,volume\,(m{m}^{3})=\frac{{w}^{2}\times l}{2}$$

where *w* = width and *l* = length, in mm, of the tumor. Tumor weight may be estimated with the assumption that 1 mg is equivalent to 1 mm^3^ of tumor volume. Eighteen days after tumor implantation, which was designated as Day 1 of the study, the animals were sorted into nine groups. The individual tumor volume of 88–172 mm^3^ and the group means tumor volume were 126–129 mm^3^.

Rexahn Pharmaceuticals, Inc. provided RX-5902-LC (code-named RX10-LC, Lot. No. RXN1084_001-4, received on 8/5/2014). CR Discovery Services assigned code names for the purpose of confidentiality during in-house testing. RX-5902-LC was protected from light and prepared by dissolving in DI water to yield 32 and 60 mg/mL dosing solutions, which provided 320 and 600 mg/kg dosages, in a dosing volume of 10 mL/kg.

Treatment began on Day 1 in all groups of mice (*n* = 11/group) with established subcutaneous MDA-MB-231 tumors. Each group was treated as indicated in the figures. All doses were adjusted per body weight. RX-5902-LC was administered orally (p.o.), once a week for 3 weeks for all regimens.

### Xenograft mouse model

SCID, female, 8-week-old mice were purchased from In-Vivos, Singapore. Mice were acclimatized to laboratory conditions for 1 week at the Animal Holding Unit, National Cancer Centre Singapore, before being employed for experiments. All experiments involving mice were performed according to protocols approved by the SingHealth Institutional Animal Use and Care Committee.

A volume of 30 µL of MDA-MB-231 Scramble and Knockdown cell inoculum (0.8 × 10^6^ cells in PBS) was injected into the mammary fat pad tissue of the mouse using 30-gauge needles (*n* = 5 for each experiment). The fat pad was surgically exposed to ensure cells were injected directly into the fat pad and not the subcutaneous space. The skin incisions were subsequently closed with wound clips. The development of tumors was monitored by quantification of the bioluminescence signals using the Xenogen IVIS system (Caliper Life Sciences, CA). Mice were euthanized when the humane endpoint criteria were met by CO_2_ inhalation. At the end of the experiment, lungs were harvested and imaged to detect the presence of metastases. Primary tumors were excised from the mice; both liver and lung tumors, and normal tissues were formalin-fixed, paraffin-embedded, for subsequent histological evaluation.

### Cell function assays

3D cell growth and cell invasion assays were performed as previously described [70]. AlamarBlue (Life Technology, CA, USA) was used to quantify the 3D Matrigel growth. For the colony formation assay, cells were allowed to grow for 8–10 days. After 10 days, cells were washed with 1 mL PBS and then fixed in 500 µL methanol for 15 min. The respective colonies were stained with 0.1% crystal violet in 20% ethanol (in PBS) for half an hour at room temperature. Photographs were taken using the UVP-GelDoc imaging system. Later colonies were dissolved in 20% acetic acid and measured at 595 nm wavelength using the Thermo-Varioskan flash plate reader.

### Mammosphere assay

Monolayer derived single cells were re-suspended in mammosphere media (Dulbecco’s modified Eagle’s medium F12 (Life Technology) supplemented with 20 ng/mL recombinant human EGF, 20 ng/mL recombinant human basic FGF, B27 supplement, 0.4% FCS, penicillin–streptomycin, L-glutamine (all from Life Technologies) and 5 µg/mL bovine insulin (Sigma) and seeded in polyhema-coated 96-well plate at a density of 2000 cells/100 µL and subsequent passages were grown as 1000 cells/100 µL and viability was measured by alamarBlue as described previously [72].

### ALDEFLUOR assay

ALDEFLUOR assay was performed as per the manufacturer’s instructions (Stem Cell Technologies, France). Flow cytometry was performed using a BD FACSCalibur™ instrument (BD Biosciences) (Cancer Sciences, University of Southampton, UK).

### CD24/CD44 cell surface markers staining

Briefly, surviving cell fractions were harvested and collected by centrifugation and resuspended in PBS with 2% FCS. 5 µL of CD24-FITC (Biolegend #382607) and 2.5 µL of CD44-APC (Biolegend #338805) were added to the respective samples and incubated for 20 min at room temperature in the dark. Respective isotype control antibodies were assayed as technical controls.

### Cell cycle analysis

MDA-MB-231 cells were transfected with scrambled siRNA or siRNA against DP103, then fixed in 70% ethanol (459844—Sigma Aldrich) overnight at 4 °C, washed twice in PBS (P5493—Sigma Aldrich), and stained for 10 min at room temperature with propidium iodide (PI)/RNase staining solution (4087—Cell Signaling Technologies). Samples were analyzed using a BD FACS Canto II cell sorter and FloJo v10 software.

### Flow cytometry for cancer stem cell characterization

MDA-MB-231-enriched cancer stem cells were grown in culture for 7 days. Cells were collected, washed twice with PBS, and stained with CD44 conjugated to allophycocyanin (CD44-APC) at 0.25 μg in 100 μL of medium and CD24 conjugated to phycoerythrin/Cy7 (CD24-PE/Cy7, Biolegend, #311119) at 0.25 μg in 100 μL of medium. Cells were incubated for 30 min in the dark before being analyzed using a BD FACS Canto II flow cytometer and FloJo v10 software.

### qRT-PCR

Total RNA was isolated from cell lines using TRIZOL reagent (Thermo Fisher Scientific, Waltham, MA, USA) following the manufacturer’s instructions. For CSCs, total intracellular RNA was extracted using ISOLATE II RNA Mini Kit (BIO-52072—Bioline, London, UK) or Reliaprep™ RNA cell miniprep system (Promega, USA) following the manufacturer’s protocol. For Drosophila, total RNA was extracted from 50 µL embryos selected for fertilization and developmental stage for each genotype using Qiagen RNA extraction kit following the manufacturer’s protocol. RNA preparations were incubated with RNAse free DNAse1 (NEB) at 37 °C for 10 min to remove any potential DNA contamination. After the treatment, DNAseI has been inactivated by incubating the RNA samples at 75 °C. RNA was quantified with a Nanodrop Spectrophotometer (Thermo Fisher Scientific, Waltham, MA, USA). RNAs with a 260/280 ratio >1.8 were used for downstream experiments.

1 µg RNA was subjected to reverse transcription using SuperScript^®^ III First-Strand System (18080051, ThermoFisher Scientific) following the manufacturer’s instructions. Real-time quantitative PCR (RT-qPCR) was carried out using either Taqman^®^ Universal PCR MasterMix (Applied Biosystems, USA) or KAPA SYBR FAST qPCR Universal Kit (#KK460) following the manufacturer’s instructions in ABI PRISM 7500 thermocycler (Applied Biosystems, Foster City, CA, USA). Normalization was performed with respect to housekeeping genes 18S or GAPDH. RPL32 was used as a reference housekeeping gene in Drosophila. Data are presented as relative expression = 2^−ΔΔCt^. Primer sequences used are listed in Table S5.

### Immunoblotting

Whole cell lysates were prepared with radioimmunoprecipitation assay (RIPA) buffer (150 mM NaCl, 1.0% IGEPAL-630, 0.5% sodium deoxycholate, 0.1% SDS, 50 mM Tris, pH 8.0) (R0278-500ML, Sigma Aldrich) with protease and phosphatase inhibitor (5872S—Cell Signaling Technologies). Protein concentration was determined using the Coomassie Protein Assay Kit (Fisher Scientific, Waltham, MA, USA).

Equal amounts of protein (30 μg) were loaded into 8–12% gels for SDS–polyacrylamide gel electrophoresis (SDS-PAGE), depending on the sizes of proteins required. The gel was then run at 80 V for 20 min for stacking of the proteins, followed by 125 V for 2 h for the resolution of the proteins, using the BioRad Mini-PROTEAN 3 Cell (CA, USA). Thereafter, proteins were transferred onto 0.45 μm nitrocellulose membrane (10600002—Amersham Protran, GE Healthcare), blocked for 1 h at room temperature with 5% skim milk powder in TBST, and washed with TBST thrice before incubating the membrane overnight at 4 °C with the primary antibody. Following three washes in TBST, secondary antibodies were incubated at room temperature for 1 h. After three washes in TBST buffer, protein bands were detected using the Supersignal West Pico Chemiluminescence (Thermo Fisher Scientific, Waltham, MA, USA), visualized with A-Plus X-Ray films (Konica Minolta) or ChemiDoc (Bio-Rad).

### Antibodies

The primary antibodies anti-DP103 (BD Biosciences, #612152)), anti-LRP6 (C5C7, #2560), anti-pLRP6 (S1490) (#2568), anti-pLRP6 (T1479), anti-c-Myc (#9402), anti-β-catenin (S33/S37/T41, #9561), anti-P-β-Catenin (S33/S37/T41) (D13A1, #8814), anti-PARP, anti-GSK3α/β (D75D3, #5676), anti-GSK3β (27C10, #9315S), anti-TCF4 (C48H11, #2569), anti-Dvl2 (Cell Signaling Technologies, #3216S)), anti-Wnt-3a (Merck Millipore, #09-162), anti-cyclin D1 (Abcam, ab134175), and α-tubulin (Santa Cruz Biotechnology, B-7, sc-5286) antibodies were diluted to a factor of 1:1000, and primary antibody anti-β-actin (Sigma-Aldrich, #A2228) and anti-GAPDH (Santa Cruz Biotechnology, sc-47724) monoclonal antibodies were diluted to a factor of 1:5000, in 1% BSA. β-actin, α-tubulin, and GAPDH antibodies were used as loading controls. For HRP-conjugated secondary antibody, affinity-purified horse anti-mouse IgG and anti-rabbit IgG were used at a dilution of 1:5000 in 1% BSA (Cell Signaling, MA, USA). Anti-Gemin3 mouse monoclonal antibody (12H12, Santa Cruz Biotechnology, TX, USA) or anti-Gemin3 rabbit polyclonal antibody (H-145, Santa Cruz Biotechnology, TX, USA) was used for IP.

### DNA and RNAi transfection

Transfections were carried out using Lipofectamine RNAiMAX (Thermo Fisher Scientific, Waltham, MA, USA) for siRNA or Lipofectamine 2000 (Thermo Fisher Scientific, Waltham, MA, USA) for DNA plasmids, according to the manufacturer’s instructions.

Two independent siRNA sequences against DP103 were purchased from Thermo Fisher Scientific (Stealth RNAi, Waltham, MA, USA) and reconstituted according to the manufacturer’s protocol. siRNA sequences as follows: siDP103#1 5’-GAU UCC UUG UCU GUC UUC CUU UAA A-3’ and siDP103#2 5’-CCA GUG AUC CAA GUC UCA UAG GUU U-3’. Non-targeting all-star siRNA (5’-GGA AAU UUG CGU GUG GAG UTT-3’) was obtained from Qiagen (Valencia, CA, USA) and used as a control. All siRNAs were used at a final concentration of 50 nM.

HA-DP103-pCMV containing full-length DP103 cDNA and empty vector HA-pCMV overexpression plasmids were purchased from Sinobiologicals (Beijing, People's Republic of China).

### Generation of DP103 shRNA stable knockdown cell lines

Customized DP103-shRNA construct (shRNA-DP103-Puro-tGFP-pLKO) was designed in the same sequence as siDP103#1. Scrambled shRNA (shRNA-scrambled-Puro-tGFP-pLKO) (Sigma-Aldrich, MO, USA) was used as a control.

Transfection of customized DP103-shRNA construct (shRNA-DP103-Puro-tGFP-pLKO) and Control scrambled shRNA (shRNA-scrambled-Puro-tGFP-pLKO) was carried out in MDA-MB-231 using third-generation lenti-viral packaging plasmid kit (Lenti-vpak packaging kit), according to the manufacturer’s protocol (OriGene Technologies, Rockville, MD, USA). Successfully infected MDA-MB-231 cells were selected with increasing puromycin concentrations (0.2–0.6 μg/mL) (Sigma) over three weeks. DP103 knockdown efficiency was confirmed by immunoblotting.

### Luciferase-based promoter activity assay

Wnt target gene c-Myc promoter activity was measured using c-Myc promoter luciferase reporter plasmid pBV-Luc/Del-1 (Addgene, Cambridge, MA, USA). Relative luciferase activity is achieved by normalizing with a thymidine kinase promoter Renilla luciferase reporter plasmid, pRLTK (Clontech, CA, USA), to control for transfection efficiency.

5 × 10^5^ cells are seeded in 24-well plates and transfected with either M50 8X Super TOPflash plasmid or M51 8X Super FOPflash plasmid (kind gifts from Professor David Marc Virshup from Duke-NUS, Singapore). Cells are also co-transfected with Renilla plasmids to control for transfection efficiency. Reporter activity was measured using the Dual Luciferase Assay System (Promega, Madison, WI, USA) as per the manufacturer’s instructions. Relative luciferase activity is calculated based on the ratio of bioluminescence readings of TOPflash normalized with Renilla against bioluminescence readings of FOPflash normalized with Renilla (TOP/FOP) and is shown as a fold difference with reference to the control. For other luciferase constructs, bioluminescence readings from the target gene promoter are represented as a ratio against Renilla bioluminescence readings to obtain relative luciferase activity.

### Co-immunoprecipitation

Cells were lysed in IP lysis buffer containing 150 mM NaCl, 1 mM EDTA, 1% glycerol, and 1% NP40, supplemented with protease inhibitor tablets (Thermo Fisher Scientific, Waltham, MA, USA). 500 μg of proteins were pre-cleared with 20 μL of pre-washed Fast Flow protein G Sepharose beads (GE Healthcare, Buckinghamshire, UK) for 1 h at 4 °C before rotating with 1.5 μg of target antibody (anti-DP103, BD Biosciences anti-GSK3β, CST or anti-β-catenin, CST) or control mouse/rabbit IgG (Merck Millipore, Darmstadt, Germany) overnight (approximately 16 h) at 4 °C and 30 μL of Fast Flow protein G Sepharose beads for pull-down. Sepharose G beads enriched IP samples were washed four times in cold IP wash buffer containing 250 mM NaCl, 1 mM EDTA, 1% glycerol, and 1% NP40, supplemented with protease and phosphatase inhibitors (Thermo Fisher Scientific, Waltham, MA, USA), at 10-min intervals. Samples were then boiled in 5X SDS loading buffer and subjected to SDS–PAGE.

### Chromatin immunoprecipitation

Cells were fixed with 1% formaldehyde at room temperature for 10 min and then neutralized with glycine for 5 min. Cells were then collected and resuspended in lysis buffer containing 50 mM Tris–HCl pH 8.0, 10 mM EDTA pH 8.0, and 1% SDS, supplemented with protease inhibitors tablets (Thermo Fisher Scientific, Waltham, MA, USA). Samples were sonicated and adjusted to similar amounts using dilution buffer containing 10 mM Tris–HCl pH 8.0, 100 mM NaCl, 1 mM EDTA, 1% Triton X-100, and 0.01% SDS, supplemented with protease inhibitors tablets (Thermo Fisher Scientific, Waltham, MA, USA). Samples were pre-cleared by incubating with Protein-G beads for 1 h, followed by addition of 2–10 μg of anti-TCF4 antibody (Cell Signaling Technologies, C48H11, #2569)) 1:50 H3K27Ac (Abcam, ab4729) or negative control IgG (Cell Signaling Technologies, MA, USA) to the supernatant. The mixture is incubated overnight with tumbling at 4 °C. The next day, the immunoprecipitates were washed 4 times with wash buffer containing 1 M Tris–HCl pH 8.0, 5 M NaCl, 0.5 M EDTA, 10% SDS, and 1% NP-40, supplemented with protease inhibitors tablets (Thermo Fisher Scientific, Waltham, MA, USA). Protein/DNA complex was eluted using an elution buffer containing 1% SDS, 0.1 M NaHCO_3_, and reverse cross-linked. DNA was purified with a PCR purification kit (Qiagen). The amount of enrichment for TCF4 in each treatment is compared relative to IgG by the comparative Ct method at the end of the qPCR run.

Primer sequences used are: DP103 Forward 5’-ACT CAC CAG TTG CCT CAT CT, DP103 Reverse 5’-GGATCTTGGGAGCGAGAAGA-3’; AXIN2 Forward 5’-GGC TCA CAC CTG TAA TCC CA-3’, AXIN2 Reverse 5’-CGC TTC CCA GGT TCA AGC TA-3’; Sp5 Forward 5’-GGG TCT CCA GGC GGC AAG-3’, Sp5 Reverse 5’-AGC GAA AGC AAA TCC TTT GAA TCC-3’, FOXC1 Enhancer Forward 5’-AGTCAGCTCCTGTGAACAGC-3’; FOXC1 Enhancer Rev 5’-CCAGCTGTCAAGGTCACCTT-3’; Gene desert Forward 5’-AACCTCACTTTCATTGTTACTAGCCATA-3’; Gene desert Rev 5’- CGCTCAAGGATGTCAGTAGCAT-3’.

### Immunofluorescence

Cells were fixed with 4% formaldehyde for 15 min at room temperature. After two washes with 1X PBS, 0.2% Triton X-100 was added to each chamber for 15 min to allow cell membrane permeabilization. Cells were next washed with 1X PBS twice. Following cell membrane permeabilization, cells were blocked with 3% BSA dissolved in PBS. After blocking, anti-DP103 (Proteintech, #11324-1-AP) and anti-GSK3β (Cell Signaling Technology, 27C10, #9315S primary antibodies were added to the cells, diluted into 1:800 and 1:500, respectively, and incubated overnight for 16 h. The next day, cells were washed twice with 1X PBS and incubated with Alexa Fluor 488 rabbit anti-mouse IgG and Alexa Fluor 594 goat anti-rabbit IgG (Invitrogen, CA, USA), diluted at 1:1000 for 2 h at room temperature with shaking, away from light. After secondary antibody incubation, cells were washed twice with 1X PBS, and glass cover slips were mounted with VECTASHIELD DAPI nuclear stain containing mounting media (Vector Laboratories) onto the cells. Confocal analysis using Olympus FluoView 1000 (FV 1000; Olympus, Japan) with identical acquisition parameters for the same image session. Pictures were analyzed with Olympus FLUOVIEW Version 1.7a Viewer.

Drosophila embryos were fixed with Heat-Methanol treatment [74] or with heptane/4% formaldehyde in phosphate buffer (0.1 M NaPO_4_, pH 7.4) [75]. The antibodies used were: anti-Armadillo (mAb N2 7A1, Developmental Studies Hybridoma Bank developed under the auspices of the NICHD and maintained by The University of Iowa, Department of Biological Sciences, Iowa City, IA 52242), anti-HA (ratAb 3F10 and mouse 12CA5, Roche), rabbit anti-Armadillo [76, 77], phospho-tyrosine pY99 (Santa Cruz Biotechnology), and anti-α-tubulin (E7, DSHB). Staining, detection, and image processing were performed as described in [78].

### Proximal ligation assay (PLA)

PLA was carried out using Duolink In Situ Detection Reagents Red kit (DUO92008, Sigma-Aldrich, France) as per the manufacturer’s instructions. Briefly, cells were seeded into Millicell EZ slide 8-well glass chamber slides (Merck Millipore, MA, USA) in complete DMEM media for 24 h. All steps were like an immunofluorescence assay, up to primary antibodies incubation: using human anti-rabbit DP103 and human anti-mouse GSK3β primary antibodies. Following primary antibody incubation, the slides were washed with Wash Buffer A, and PLA probes (1:5 in antibody diluent) were added, and the slides were incubated in a pre-heated humidity chamber for 1 h at 37 °C. Next, the ligation stock was diluted 1:5 in high-purity water mix and added to the chamber slides after washing with wash buffer A and incubated for 30 min at 37 °C. Later, amplification was done using amplification-polymerase solution (1:5 in high-purity water) and incubated for 100 min at 37 °C. Amplification-polymerase solution was tapped off, and slides were washed with 1X wash buffer B for 2–10 min. Let the slides dry at room temperature in the dark, and the images were observed under a confocal microscope.

### Stable isotope labeling with amino acids in cell culture (SILAC) study of DP103 interactome

MDA-MB-231 cells were stably transfected with sh-DP103 or sh-Scramble, followed by puromycin selection before SILAC. These stable cells were cultured in DMEM for SILAC (Thermo Fisher Scientific) supplemented with 10% dialyzed FBS and 1% Penicillin/Streptomycin containing either normal isotopes of L-lysine-(12C614N2) (K0) and L-arginine-(12C614N4) (R0) (K0R0-‘light’medium) for shDP103 or stable isotope L-lysine-(13C615N2) (K8) and L-arginine-(13C615N4) (R10) (R10K8-‘heavy’ medium) in the case of shScramble cells. The cells were cultured in Heavy or Light medium for at least six doublings to ensure efficient incorporation of labeled amino acids. The whole process of SILAC was performed as described (Chakraborty et al., 2015). Briefly, cells from an 8 × 15 cm dish were lysed in IP lysate buffer (Pierce™ IP Lysis Buffer, #87788) with the addition of proteinase inhibitor (Sigma, #P8340), phosphatase inhibitor (Sigma, #P0044), Pierce Universal Nuclease (Pierce, #88701), and DTT (Sigma, #43815). 80 µL Magnetic beads (Dynabeads™ Protein G Immunoprecipitation Kit, Thermofisher #10007D) were washed with IP lysate buffer and coupled with 40 µg DP103 antibody (Santa Cruz, #sc50405) overnight at 4 °C. 30 mg of total lysates from Heavy or Light medium labeled cells were pre-cleaned by 20 µL magnetic beads each for 1 h, followed by IP with 40 µL antibody-coated beads for 4 h at 4 °C. Following IP, the samples were washed for 4 times with IP Lysate buffer and eluted in 30 µL 1.5X NuPAGE LDS sample buffer with 0.1% DTT. Eluted samples were heated at 95 °C for 10 min. The IP elution was separated by a one-dimensional NuPAGE™ 4–15% Bis–Tris Protein Gels, stained with Colloidal Blue (Invitrogen). Protein bands were cut and digested with trypsin. The samples were further analyzed on an Orbitrap Classic (Thermo Fisher Scientific). Identification and quantification were performed using MaxQuant version 1.5.0.30.

### Bio-layer interferometry

Biolayer Interferometry (FortéBIO Octet RED 96 instrument, PALL Corporation) was used to study the interactions between GSK3β and Axin1 peptide or predicted interacting DP103 peptides. Peptides were diluted in assay buffer (50 mM Tris, pH 7.4, 0.1% BSA, 0.1% Tween-20). To characterize the protein–peptide binding, streptavidin-coated biosensors were preloaded with 60 μg/mL biotinylated peptides for 250 s and then immersed in different concentrations of GSK3β protein (0, 2, 5, 7.5, 10, 15, and 20 μg/mL) for 300 s association, followed by 300 s dissociation in assay buffer. The buffer control was subtracted to account for the background. The Octet software, v 9.0.0.14 (PALL), was used to analyze the binding data. Nonlinear regression fitting using a 1:1 binding mode was used to derive the dissociation constants.

### Data preprocessing for gene expression

A meta-cohort of human breast cancer on Affymetrix U133A or U133Plus2 platforms was curated and compiled previously [76]. Briefly, microarray data were downloaded from Gene Expression Omnibus (GEO). Robust multichip average (RMA) normalization was performed on each dataset. The normalized data were combined for each disease and subsequently standardized using ComBat [77] to remove batch effects. This meta-cohort of human breast cancer consists of 3992 samples from 26 cohorts.

To obtain the molecular subtype of breast cancer for the 3992 breast cancer samples, we employed single-sample Gene Set Enrichment Analysis (ssGSEA) [78] to compute, for each sample, enrichment scores of breast cancer subtype signature (Basal, Claudin-low, Luminal-A, Luminal-B, ERBB2, or Normal-like) [4]. Each sample was then assigned as the subtype with the highest enrichment score.

Statistical significance evaluation by the Mann–Whitney test and the Spearman correlation test was computed using Matlab® 2016b. Log-rank test, Kaplan–Meier, and dot plots were performed in Graphpad Prism 6.

### Mutation and copy number aberration profile of DP103

The mutation and copy number aberration profiles of breast invasive carcinoma and metastatic breast cancer were queried on cBioPortal version 1.10.1 [79, 80]. Only cohorts with both mutation and GISTIC copy number aberration results were considered.

### Bioinformatics analysis of cohort datasets

#### Data analysis and collection

Breast carcinoma datasets were obtained from the TCGA and METABRIC studies via cBioPortal in November 2017; specifically, the Breast Invasive Carcinoma (TCGA, Provisional) Breast Cancer (METABRIC) data sets. Examination and enrichment of gene families was facilitated via Perl scripts developed in-house.

#### Gene set enrichment analysis

Gene set enrichment analysis (GSEA) was performed using the GSEA software. TNBC subtypes were selected on the criteria of being ER^−^/PR^−^/HER2^−^ by immunohistochemistry (IHC). The list of patients corresponding to each patient group for comparison was manually provided to GSEA to create phenotypes for analysis. GO gene sets were used in the analysis. Gene sets affording *p*-values < 0.05 and *q*-values (false discovery rates) of <0.25 were selected as being significantly altered between the patient groups.

Statistical significance evaluation by the Mann–Whitney test and the Spearman correlation test was computed using Matlab® 2016b. Log-rank test, Kaplan–Meier, and dot plots were performed in Graphpad Prism 6.

### Molecular docking analysis

The X-ray crystal structure of the GSK3β–Axin complex bound to the phosphorylated LRP6 c-motif (PPP-pT-PR) (PDB code 4NM5) [41] provided the GSK3β structure for the modeling studies. The structure was prepared using the Protein Preparation Wizard. Crystallographic waters were removed, and missing sidechains were added. Sidechains with multiple crystallographic conformations were set to either the conformation with the greatest occupancy or the conformation with greater hydrogen bonding. The Axin molecule was removed from the structure.

Phosphorylation sites in DP103 were identified from UniProt (accession number Q9UHI6). Structures of DP103-derived hexapeptides containing the phosphosites were prepared by in silico mutation of the phosphorylated LRP6 c-motif structure derived from its co-complex with GSK3β. DP103-derived hexapeptides all featured phosphosites at the fourth residue from the N-terminus, as in the bound LRP6 c-motif.

Molecular docking was performed using Glide 6.8. A receptor grid suitable for peptide docking to GSK3β was generated at the centroid of the bound LRP6 phosphopeptide. Positional constraints at the locations of the phosphate (0.5 Å sphere) and the N-terminus (2.0 Å sphere) of the bound LRP6 peptide were specified during receptor grid generation. These constraints were defined so as to ensure that the phosphate of each peptide docked to the same position, as well as to ensure a consistent N-to-C direction of binding by all peptides. All other options for receptor grid generation were kept as defaults. The 16 DP103-derived phosphopeptides, as well the bound LRP6 phosphopeptide and the GSK3β-derived autoinhibitory peptide (RTT-pS-FA) were docked to GSK3β. The SP-Peptide mode was employed for ligand docking. Ring conformations were not sampled during ligand docking, and only trans amide conformations were allowed. Only poses satisfying all the constraints specified during receptor grid generation were permitted to be returned. Up to 100 poses per ligand are subject to post-docking minimization. All other ligand-docking options were kept as defaults. Using this docking protocol, root-mean-squared deviations (RMSD) of <2.0 Å between the structures of the docked and crystallized LRP6- (RMSD 0.9 Å relative to visible portion of bound peptide in PDB 4NM5) and GSK3β-derived (RMSD 1.7 Å relative to visible portion of bound peptide in PDB 4NM3) peptides, thus validating the protocol’s ability to generate realistic structures. Binding energies for the best-scoring poses of each peptide were then predicted using Prime MM-GBSA 3.000. These calculations used the VSGB solvation model and the OPLS3 force field (the default settings for these parameters). In addition to the peptide, GSK3β residues within 6.0 Å of the peptide were sampled by minimization.

### Molecular dynamics simulations and binding energy calculations

Selected GSK3β–peptide complexes following molecular docking were subject to molecular dynamics (MD) simulations using GROMACS 2020.3 [79], followed by per-residue decomposed molecular mechanics-generalized Born/surface area (MM-GB/SA) calculations using the MMPBSA.py facility [80] within AmberTools [81]. Systems for simulation were parameterized using the ff14SB force field for proteins and peptides [82], ff14SB-associated parameters for phosphorylated amino acids [83], ion-oxygen distance-optimized parameters for magnesium in TIP3P water [84], and previously prepared AMBER force field-compatible parameters for ADP [85]. Topologies were converted to GROMACS format using acpype [86], with subsequent system preparation and simulation taking place in GROMACS 2020.3. GSK3β–peptide complexes were placed in boxes of TIP3P water [87] with a minimum distance of 1.5 nm to the box edge, and charge-neutralized by the addition of chloride ions [88]. Solvated and charge-neutralized systems were equilibrated in the NVT and NPT ensembles [89, 90], and up to five 50 ns simulations of each protein–peptide complex at 298 K and 1 atm were performed. The root-mean-squared deviation (RMSD) in the atomic coordinates over time of each simulation was followed, and three simulations exhibiting minimal variation in this measure over the final 10 ns of the simulation trajectory were selected for MM-GB/SA analysis. For each protein-peptide complex, MM-GB/SA analysis was conducted using the final 10 ns of the three simulation trajectories, and the results were averaged from these using in-house scripts. The Onufriev–Bashford-Case GB model (igb = 5) [91] and the LCPO method [92] were used to compute the polar and nonpolar desolvation components of the binding energy, respectively. Per-residue decomposition (idecomp = 1) was employed to investigate the magnitude to which specific peptide residues contributed to binding.

### H3K27ac ChIP-seq

H3K27ac ChIP-seq data of TNBC cells were downloaded from the European Nucleotide Archive (ENA; accession number PRJEB33558 [93]). They were mapped to the human hg19 genome by bowtie2 [94] (version 2.3.5) with default parameters. MACS2 [95] (version 2.0.10.2014XXXX) was used to call ChIP-seq peaks using default settings. The resulting bedGraph files were converted to BigWig through bedGraphToBigWig (version 4). All data was visualized through uploading BigWig files to the UCSC genome browser to build custom tracks. The BigWig file of H3K27ac ChIP exo seq data of TNBC cells was downloaded from the Gene Expression Omnibus (GEO; accession number GSE118033 [96]) and uploaded to the UCSC genome browser to visualize. TCF7L2 ChIP-seq of 7 cell lines from ENCODE [97, 98] at the DDX20 gene region, which is highlighted in blue.

### Generation and maintenance of gastric cancer patient-derived organoids

The study was approved by the local ethics board (National Healthcare Group, Domain Specific Review Board Ref Nos. 2016/00059 and 2019/00924). Patients diagnosed with gastric adenocarcinoma and undergoing surgical resection or endoscopy at the National University Hospital, Singapore, were enrolled after written informed consent was obtained. Human gastric tumor tissues were biopsied from each gastric cancer patient during the procedure and processed as described previously [99]. Briefly, tissues were first minced and washed in Dulbecco’s phosphate-buffered saline (DPBS; Thermo Fisher Scientific, Waltham, MA), and then digested in DPBS containing 1 mg/mL collagenase (Sigma-Aldrich, Saint Louis, MI) and 2 mg/mL bovine serum albumin (BSA; Sigma-Aldrich) for 30 min at 37 °C. Digested tissues were passed through 30 μm filters (Miltenyi Biotec, Bergisch Gladbach, Germany). Filtered cells were pelleted at 300×*g* for 5 min, resuspended in Matrigel (Corning Life Sciences, Corning, NY), and seeded into multiwell plates (Thermo Fisher Scientific). Cultures were maintained in culture medium containing growth factors at 37 °C in 5% CO_2_ and monitored daily for organoid generation.

### Pathway analysis using GSEA

Differentially expressed gene (DEG) analysis was performed between DP103 high vs DP103 low samples using DESeq2 v1.46.0, followed by LFC shrinkage using ashr v2.2-63. The DEGs were next used for GSEA analysis through the clusterProfiler v4.14.4 package on GO, KEGG, Hallmark, and Reactome pathways from msigdb (msigdbr v24.1.0). NES scores and adjusted p-values from Wnt-related pathways were manually selected and shown as bar plots.

### Correlation analysis between DP103 and Axin2 in GC organoid samples

TPM values of DP103 and Axin2 from 15 gastric cancer organoid bulk RNA-seq samples were used for performing Pearson’s correlation analysis using R v4.2. The 15 samples include Q10TE, Q13TE, Q15TE, Q16TE, Q24TE, Q62TE, Q6TE, S46TE, S47TE, S54TE, S73TE, S74TE, S78TE, S86TE, and SN6TE.

### Three-dimensional TNBC tumor spheroid culture and drug response

A total of 10,000 MDA-MB-231 cells in 100 µL of DMEM per well were seeded in ultra-low attachment (ULA) plates (Corning) and cultured for 3 days to form tumor spheroids. Independent dose titration assays were performed by exposing spheroids to graded concentrations of Olaparib (1–30 µM) or RX-5902 (10–100 nM). Vehicle controls received 0.1% DMSO. Spheroid morphology was monitored by confocal microscopy, with images acquired at 0, 24, and 48 h of treatment. At 48 h post-treatment, spheroids were harvested by gravity sedimentation for 5 min. Eight spheroids were pooled per sample (*n* = 6), and trypsinization was performed for 1 min prior to performing cell viability and total cell count analysis using Countess II FL automated cell counter (Thermo Fisher Scientific). Dose–response curves and the half-maximal inhibitory concentration (IC₅₀) were determined by logistic non-linear regression model using GraphPad Prism 10. Based on the IC₅₀ values obtained, drug combination treatments were subsequently conducted for 72 h. Spheroid morphology during the combination treatment was imaged at 0, 24, 48, and 72 h using confocal microscopy. Following the 72-h treatment, spheroids were harvested, and viability assays were performed as described above.

### Statistical analysis for in vitro assays

Statistical analyses for all in vitro experiments were performed using the SPSS package or in GraphPad Prism (version 15.0 for Windows, SPSS Inc., USA). Respective cluster bar graphs and line graphs were created using GraphPad Prism (La Jolla, CA, USA). The Student’s *t*-test analysis was performed with significance set at the 5% level (two-tailed).

## Supplementary information

Supplementary File

Figure S1

Figure S2

Figure S3

Figure S4

Figure S5

Figure S6

Figure S7

Supplementary Table S1

Original Data

## Data Availability

Data are available from the corresponding author on reasonable request.

## Code availability

New code generated in this study is available on reasonable request from the authors.
