# Supplementary material for: DP103 as a critical modulator of Wnt signaling and cancer stemness: implications for precision treatment in triple negative breast cancer
Source: Cell Death Dis. 2026 Jun 5;17(1):673. doi: 10.1038/s41419-026-08943-3 (PMC13434658; doi:10.1038/s41419-026-08943-3)
Supplement: Supplementary file 1 [file 41419_2026_8943_MOESM1_ESM.docx]

**Supplemental Information**

**DP103 as a Critical Modulator of Wnt Signaling and Cancer Stemness: Implications for Precision Treatment in Triple Negative Breast Cancer**

Wanpei Cai^1,2,33^, Sebastian Öther-Gee Pohl^3,33^, Xianning Lai^1,2,33^, Hiu Yan Lam^1,2^, Catharine Shi Yun Low^4^, Vivien Koh^5,6^, Haoran Ma^7^, Mark Agostino^8^, Arindam Banerjee^9,10^, Nawat Bunnag^1,2,11^, Jahnavi Suresh^7,11^, Amudha Deivasigamani^12^, Yi Yuan^1,2^, Tuan Zea Tan^6^, Muthukumar Rajasekaran^13^, Jianxiang Chen^12^, Hannah Lee Foon Swa^14^, San Hue Hua^15^, Shiyuan Guo^15^, Janet Cheng-Fei Teng^15^, Jas Min Chin^15^, Jocelyn Yeen Hui Choy^6^, Aye Aye Thike^16^, Young Bok Lee^17^, Madhu Mathi Kanchi^1,2,18^, Karthik Ramesh^19^, Yu Keung Mok^19^, Ruby Yun-Ju Huang^20^, Jayantha Gunaratne^21^, Melissa Jane Fullwood^6,22^, Puay Hoon Tan^16,23^, George W Yip^15^, Gautam Sethi^1,2^, Jeremy P. Blaydes^9^, Celestial T. Yap^24^, Peter Edward Lobie^1,25^, Venetia Jing Tong Kok^4^, Arunasalam Dharmarajan^26,27,28^, Kam Man Hui^29^, Deog Joong Kim^17^, Jit Kong Cheong^2,4^, Nicholas Tolwinski^7,11^, Wei Peng Yong^5,6^, Patrick Tan^7,24,30,31,32^, Kevin B. Myant^3^, Alan Prem Kumar^1,2,^*

**Authors affiliations:**

^1^Department of Pharmacology, Yong Loo Lin School of Medicine, National University of Singapore, Singapore

^2^NUS Centre for Cancer Research (N2CR), Yong Loo Lin School of Medicine, National University of Singapore, Singapore

^3^Cancer Research UK Edinburgh Centre, Institute of Genetics and Cancer, The University of Edinburgh, Western General Hospital Campus, Crewe Road, Edinburgh, EH4 2XU, Scotland.

^4^Department of Biochemistry, Yong Loo Lin School of Medicine, National University of Singapore

^5^National University Cancer Institute Singapore, National University Health System, Singapore

^6^Cancer Science Institute of Singapore, National University of Singapore, Singapore

^7^Program in Cancer and Stem Cell Biology, Duke-NUS Medical School, Singapore 169857, Singapore

^8^Curtin Institute for Computation, Curtin University, Perth, Australia

^9^Cancer Sciences Unit, Faculty of Medicine, University of Southampton, United Kingdom

^10^Biocon Limited, SEZ Unit Biocon Park, Bangalore, India.

^11^Division of Science, Yale-NUS College, Singapore

^12^Division of Cellular and Molecular Research, National Cancer Centre Singapore, Singapore

^13^Programme in Cardiovascular & Metabolic Disorders, Duke-NUS Medical School, Singapore

^14^Diagnostics Development Hub, A*STAR, Singapore

^15^Department of Anatomy, Yong Loo Lin School of Medicine, National University of Singapore, Singapore

^16^Division of Pathology, Singapore General Hospital, Singapore

^17^Rexahn Pharmaceuticals, Inc., Rockville, Maryland, USA

^18^Department of Genome Sciences, The John Curtin School of Medical Research, The Australian National University, Canberra, ACT 2601, Australia

^19^Department of Biological Sciences, National University of Singapore, Singapore

^20^School of Medicine, College of Medicine, National Taiwan University, Taipei, Taiwan

^21^Institute of Molecular and Cell Biology, A*STAR, Singapore

^22^School of Biological Sciences, Nanyang Technological University, Singapore, Singapore, Singapore

^23^Luma Medical Centre, Singapore

^24^Department of Physiology, Yong Loo Lin School of Medicine, National University of Singapore, Singapore

^25^Tsinghua Berkeley Shenzhen Institute, Tsinghua University Graduate School at Shenzhen, Shenzhen, China

^26^Trichy SRM Medical College Hospital and Research Centre

^27^School of Human Sciences, Faculty of Life and Physical Sciences, The University of Western Australia, Perth, Australia

^28^Curtin Medical School, Curtin University, Perth, Western Australia, Australia

^29^Micro Tech Health Sdn Bhd, Kuala Lumpur, Malaysia

^30^Genome Institute of Singapore, Agency for Science, Technology and Research, Singapore

^31^Singapore Gastric Cancer Consortium, Singapore

^32^Singhealth/Duke-NUS Institute of Precision Medicine, National Heart Centre Singapore, Singapore

^33^These authors contributed equally

***Corresponding author:**

Alan Prem Kumar, PhD

Department of Pharmacology, Yong Loo Lin School of Medicine, National University of Singapore, MD3, 16 Medical Drive, Level 4, #04-01W, Singapore 117600. Phone: [+65-6516 4516](mailto:+65-6516%204516); Fax: [+65-6873 7690](tel:+6568737690) ; Email: [apkumar@nus.edu.sg](mailto:apkumar@nus.edu.sg)

**Supplementary Figure Legend**

**Figure S1. DP103 regulates Wnt pathway through PTM of Wnt modulators (Related to Figure 1 and 2).**

(A) OncoPrint extracted from cBioPortal of genomic alterations in breast cancer samples.

(B) Correlation plot of the expression of DP103 and Axin2 in GC organoid samples. Each dot represents an organoid sample, where x-axis represents DP103 TPM expression and y-axis represents Axin2 TPМ expression. The red line denotes a linear regression of the dots.

(C) Barplot showing the GSEA analysis result between DP103 high vs DP103 low using Hallmark, KEGG, GO and Reactome databases. The length of the bar represents NES values, and the colour of the bar represents -log10 (adjusted p-values).

(D-E) Effects of Wnt stimulation by GSK3α/β inhibitor (BIO) and Wnt-3a conditioned media (CM) on nuclear localisation of β-catenin in TNBC cells.

(F) Western blot analysis comparing protein expressions of DP103 and Wnt receptor LRP6. Quantification reflects pLRP6 S1490 protein levels normalised to tubulin protein levels.

**Figure S2. Establishment of cancer tumor models in Drosophila (Related to Figure 2).** (A) Schematic of the Drosophila Intestinal Epithelium which consists of two functional cell types, absorptive cells (Enterocytes, EC) and secretory cells (Enteroendocrine cells (EE). The epithelium is maintained by intestinal stem cells (ISC) that divide symmetrically to make more ISCs or asymmetrically to create transit amplifying cells (Enteroblasts, EB) which divide to restore the epithelium. ISCs are identified by their expresion of the gene escargot, here tagged with GFP. ISCs are small cells residing below the gut epithelium and contain only a single chromosome complement, unlike ECs that undergo endoreplication.

(B) (Top-left) Control esg>GFP midguts (Bottom-left) ΔArm expression under esg-Gal4 control leading to highly proliferative stem cells and enlarged gut diameter, similar to (Right) UAS-RasV12, a well-studied oncogenic transgene where Ras is constitutively active. Graph show quantification of gut width in the two overproliferation models compared to wild-type. Five independent flies per condition were dissected for the quantification. Presented is the data from one representative experiment of three.

(C) Flies expressing oncogenic constructs display hyperproliferative intestinal stem cells as shown by the increase in phosphorylated Histone 3 staining with the total number of positive cells quantified in (D). Images shown are representative of a minimum of three experiments.

(E) qPCR analysis of Gemin3 mRNA in control versus Gemin3-overexpressed flies.

**Figure S3. DP103 interacts with GSK3β (Related to Figure 3).**

(A) Root-mean-squared deviation (RMSD) over time for each of the three simulations of GSK3β complexes with the four peptides examined - the GSK3β autoinhibitory peptide (A), LRP6 c-motif (B), DP103-derived SVQ-pT-PV (C), DP-103-derived SES-pT-PV (D). RMSD calculated for the peptide relative to the starting coordinates following optimal overlay of the protein over the trajectory.

(E) Plot of per-residue decomposed binding energies against amino acid position for the four peptides examined - the GSK3β autoinhibitory peptide (blue), LRP6 c-motif (red), DP103-derived SVQ-pT-PV (orange), DP-103-derived SES-pT-PV (gray).

**Figure S4. DP103 is required for CSC formation, a downstream effect of Wnt/β-catenin pathway (Related to Figure 4).**

(A) Heatmap showing genes deregulated upon DP103 depletion in MDA-MB-231 cells.

(B) MDA-MB-436 control and DP103 siRNA knockdown cells were re-seeded in triplicates in 3D matrigel, serially diluted into 1000, 500 and 250 cells per 96-well. Scale bar of representative pictures shown is 100µm. Quantification of percentage cell growth was carried out using alamarBlue cell viability assay, where absorbance of DP103 siRNA knockdown cells were compared against control cells, normalized to 100% ± SD, n = 3. * p < 0.05, *** p< 0.001.

(C) Percentage mammosphere formation in MDA-MB-436 CSCs transfected with control siRNA or siRNA against DP103.

(D) Quantitation of cell cycle analysis performed on MDA-MB-231 CSCs 48 hours after transfection with either control siRNA or siRNA directed at DP103. At 48 hours post transfection cells were fixed and stained with PI containing RNase and analysed by flow cytometry. Results represent the mean ± SD. n = 3. * p < 0.05.

(E) Representative cell cycle histograms of quantification of MDA-MB-231 CSCs after cell cycle analysis.

(F) Gene set enrichment analysis (GSEA) on the TNBC subset of the METABRIC cohort.

(G) Quantification of DP103 levels in MDA-MB-231 CSCs infected with control shRNA or shRNA against DP103. Results represent the mean ± SD. n = 3. *** p < 0.001.

(H) Protein levels of DP103 in MDA-MB-231 CSCs infected with control shRNA or shRNA against DP103.

(I) ALDH1 stem cell marker expression in MDA-MB-436 control and DP103 siRNA knockdown TNBC cells were quantified using ALDEFLUOR Assay. Percentage of DEAB cells (negative control) were gated at 0%. Percentage of ALDH1 stained population of control and DP103 siRNA knockdown cells were normalized against its respective negative control DEAB cells to obtain percentage ALDH1 positive cells.

**Figure S5. Depletion of DP103 reduces tumor growth and metastasis in vivo (Related to Figure 4).**

(A) Representative photomicrographs to illustrate the immunohistochemical staining of DP103 in the Scramble and Knockdown samples.

(B-C) Primary tumor and lung metastases evaluated using total photon counts.

(D) Orthotopic tumor burden of MDA-MB-231 Scramble and MDA-MB-231 Knockdown at day 30, p-value = 0.0386 (unpaired T-test).

(E) Lung metastasis tumor burden of MDA-MB-231 Scramble and MDA-MB-231 Knockdown at day 30.

**Figure S6. DP103 expression is regulated by the Wnt pathway (Related to Figure 5).**

(A) TCF7L2 ChIP-seq of 7 cell lines from ENCODE at the DP103 (DDX20) gene region (highlighted in blue).

(B) H3K27Ac ChIP-seq of 3 cell lines (HCC1419, HCC1806, and HCC202) from published data at the DDX20 gene region (highlighted in blue).

(C-F) Effects of Wnt/β-catenin stimulation by Wnt-3a CM and GSK3α/β inhibitor (BIO) on DP103 mRNA protein expression in MDA-MB-436. Graphs represent the mean ± SD. n=3. *p< 0.05, **p< 0.05, **p<0.001.

**Figure S7. RX-5902 reduces the survival of TNBC cells (Related to Figure 6).**

(A) Gene expression of DP103 in and MDA-MB-436 cells treated with either RX-5902 or RX-5433 for 18 hours. Values were calculated by the 2^-ΔΔCT^ method relative to GAPDH. Results represent the mean ± SD. n = 3. * p < 0.05, ** p < 0.01.

(B) Protein expression of DP103 in MDA-MB-436 cells treated with either RX-5902 or RX-5433. Tubulin was used as a loading control.

(C) TCF luciferase activity in MDA-MB-436 cells treated with either RX-5902 or RX-5433. Results represent the mean ± SD. n = 3. * p < 0.05, ** p < 0.01.

(D) TCF luciferase activity in MDA-MB-436 cells treated with RX-5902 relative to DMSO.

(E) Treatment of RX-5902-LC results in a dose-dependent decrease in Wg mRNA expression in adult fly midguts. Results represent the mean ± SD. n = 3. * p < 0.05.

(F-G) Intestinal organoids of wild-type, Apc-/- (Wnt activated) and KPN (high metastatic potential) were treated with 50, 100 and 200 nM RX-5902. Graph represent the mean ± SD. n = 3. ** p < 0.01, *** 0.001.

(H) Cell viability of MDA-MB-231 tumor spheroids after 72-hour treatment with vehicle control, Olaparib (23.24 µM), RX-5902 (23.84 nM), or the combination of Olaparib and RX-5902. Results represent the mean ± SD. n = 9. **** p < 0.0001.

Table S2 (Related to Figure 3). Binding energies calculated for all DP103-derived phosphopeptides.

| **Peptide** | **Residue numbers** | **Binding energy (kcal/mol)** | **Binding energy difference relative to GSK3β peptide (kcal/mol)** |
| --- | --- | --- | --- |
| SVQ-pT-PV^a^ | 549-554 | -95.7 | -11.5 |
| SES-pT-PV^a^ | 685-690 | -76.2 | +8.0 |
| DLS-pS-PR | 45-50 | -75.7 | +8.5 |
| AVG-pS-PG | 184-189 | -73.0 | +11.2 |
| GSD-pT-PN | 702-707 | -70.4 | +13.8 |
| SGL-pS-VK | 502-507 | -66.2 | +18.0 |
| EGS-pS-DN | 675-680 | -65.7 | +18.5 |
| RNN-pS-VS | 497-502 | -59.3 | +24.9 |
| SGD-pS-ES | 649-654 | -58.7 | +25.5 |
| GNK-pS-YL | 669-674 | -56.8 | +27.4 |
| YQE-pS-PG | 711-716 | -52.2 | +32.0 |
| ESD-pS-DS | 653-658 | -48.6 | +35.6 |
| KAT-pS-PK | 529-534 | -45.3 | +38.9 |
| LEG-pS-SD | 674-679 | -38.6 | +45.6 |
| PNG-pS-DT | 700-705 | -28.7 | +55.5 |
| DSE-pS-DS | 651-656 | -20.3 | +63.9 |

^a^Data for these peptides is shown in Table S3, but also included here for clarity and comparison.

Table S3 (Related to Figure 3). Binding energies calculated for the binding of selected phosphopeptides to GSK3β.

| **Peptide** | **Derived from** | **Residue numbers** | **Binding Energy (kcal/mol)** | **Binding energy difference relative to GSK3β peptide (kcal/mol)** |
| --- | --- | --- | --- | --- |
| SVQ-pT-PV | DP103 | 549-554 | -95.7 | -11.5 |
| PPP-pT-PR | LRP6 | 1569-1574 | -86.6 | -2.4 |
| RTT-pS-FA | GSK3β | 6-11 | -84.2 | 0.0 |
| SES-pT-PV | DP103 | 685-690 | -76.2 | +8.0 |

Table S4 (Related to Figure 3). Binding energies of peptides with GSK3β from MD-based MM-GB/SA.

| **Peptide** | **ΔG_vdw_^1^** | **ΔG_ele_^2^** | **ΔG_GB_^3^** | **ΔG_SA_^4^** | **ΔG^5^** |
| --- | --- | --- | --- | --- | --- |
| GSK3β autoinhibitory peptide | -52.3 ± 0.1 | -525.5 ± 0.6 | +503.1 ± 0.6 | -8.5 ± 0.0 | -83.1 ± 0.2 |
| LRP6 c-motif | -50.0 ± 0.1 | -540.6 ± 0.6 | +517.6 ± 0.5 | -7.6 ± 0.0 | -80.5 ± 0.2 |
| DP103 SVQ-pT-PV | -52.9 ± 0.1 | -709.2 ± 1.2 | +672.0 ± 1.0 | -8.4 ± 0.0 | -98.6 ± 0.2 |
| DP103 SES-pT-PV | -43.1 ± 0.1 | -863.7 ± 1.0 | +835.4 ± 0.9 | -7.3 ± 0.0 | -78.8 ± 0.2 |

^1^Molecular mechanics van der Waals term. ^2^Molecular mechanics electrostatics terms. ^3^Polar desolvation from generalized Born model. ^4^Nonpolar desolvation from LCPO model. ^5^Binding energy, as given by the sum of the preceding component terms.

Table S5 (Related to Methods). qPCR primers used in the study.

| **Gene name** | **Primer (5ʹ - 3ʹ)** |
| --- | --- |
| DP103 Fwd | ATGGAAGGCTTAGAGTGTCA |
| DP103 Rev | TGGCAGGCAAGGAAGAATAAA |
| GAPDH Fwd | TCAAGAAGGTGGTGAAGCAG |
| GAPDH Rev | TCGCTGTTGAAGTCAGAGGA |
| DP103 Fwd | GCACAGCAGAGCACAACATT |
| DP103 Rev | GCATCAATCCCACGAGAAGT |
| RNF43 Fwd | GCACTTGGTTTGGTTGCAGA |
| RNF43 Rev | GTAGCATTCCTGTTGCGCTG |
| ABCG2 Fwd | CACAGGTGGAGGCAAATCTT |
| ABCG2 Rev | CCGAAGAGCTGCTGAGAACT |
| AXIN2 Fwd | TAACCCCTCAGAGCGATGGA |
| AXIN2 Rev | CCTCCTCTCTTTTACAGCAGGG |
| FZD7 Fwd | CGACGCTCTTTACCGTTCTC |
| FZD7 Rev | GCCATGCCGAAGAAGTAGAG |
| SOX2 Fwd | CCATCCACACTCACGCAAAA |
| SOX2 Rev | TATACAAGGTCCATTCCCCCG |
| POU5F1 Fwd | TCCCATGCATTCAAACTGAGG |
| POU5F1 Rev | CCAAAAACCCTGGCACAAACT |
| NANOG Fwd | TGGACACTGGCTGAATCCTTC |
| NANOG Rev | CGTTGATTAGGCTCCAACCAT |
| KLF4 Fwd | CTGCGGCAAAACCTACACAA |
| KLF4 Rev | GGTCGCATTTTTGGCACTG |
| GAPDH Fwd | CAGAACATCATCCCTGCCTCTACT |
| GAPDH Rev | GTCGCTGTTGAAGTCAGAGGAGAC |
| 18S Fwd | gcaattattccccatgaacg |
| 18S Rev | gggacttaatcaacgcaagc |
| wg Fwd | ACCCAACAACATTACGCCCA |
| wg Rev | CCAGTACACCGGGATTGTCC |
| en Fwd | ACAGCCAAAGGACAAGACCAA |
| en Rev | TCTCCGCTCGGTCAGATAGC |
| dGem3 Fwd | TAAGACCTTCGAGGAACTGCG |
| dGem3 Rev | CGTCACGAAATTGTTGCGCT |
| RpL32 Fwd | GCCCAAGGGTATCGACAACA |
| RpL32 Rev | GCGCTTGTTCGATCCGTAAC |
